# Pseudouridines on *Trypanosoma brucei* spliceosomal small nuclear RNAs and their implication for RNA and protein interactions

**DOI:** 10.1093/nar/gkz477

**Published:** 2019-05-31

**Authors:** K Shanmugha Rajan, Tirza Doniger, Smadar Cohen-Chalamish, Dana Chen, Oz Semo, Saurav Aryal, Efrat Glick Saar, Vaibhav Chikne, Doron Gerber, Ron Unger, Christian Tschudi, Shulamit Michaeli

**Affiliations:** 1 The Mina and Everard Goodman Faculty of Life Sciences and Advanced Materials and Nanotechnology Institute, Bar-Ilan University, Ramat-Gan 52900, Israel; 2 Sheba Medical Center, Tel-HaShomer 5265601, Israel; 3 Departmentof Epidemiology and Microbial Diseases, Yale School of Public Health, New Haven, CT 06536, USA

## Abstract

The parasite *Trypanosoma brucei*, the causative agent of sleeping sickness, cycles between an insect and a mammalian host. Here, we investigated the presence of pseudouridines (Ψs) on the spliceosomal small nuclear RNAs (snRNAs), which may enable growth at the very different temperatures characterizing the two hosts. To this end, we performed the first high-throughput mapping of spliceosomal snRNA Ψs by small RNA Ψ-seq. The analysis revealed 42 Ψs on *T. brucei* snRNAs, which is the highest number reported so far. We show that a trypanosome protein analogous to human protein WDR79, is essential for guiding Ψ on snRNAs but not on rRNAs. snoRNA species implicated in snRNA pseudouridylation were identified by a genome-wide approach based on ligation of RNAs following *in vivo* UV cross-linking. snRNA Ψs are guided by single hairpin snoRNAs, also implicated in rRNA modification. Depletion of such guiding snoRNA by RNAi compromised the guided modification on snRNA and reduced parasite growth at elevated temperatures. We further demonstrate that Ψ strengthens U4/U6 RNA–RNA and U2B"/U2A’ proteins-U2 snRNA interaction at elevated temperatures. The existence of single hairpin RNAs that modify both the spliceosome and ribosome RNAs is unique for these parasites, and may be related to their ability to cycle between their two hosts that differ in temperature.

## INTRODUCTION

The most abundant modifications on ribosomal RNA (rRNA) are 2′-*O*-methylation (Nm) and pseudouridinylation (Ψ). These modifications are guided by the C/D or H/ACA small nucleolar RNAs (snoRNAs), which guide Nm and Ψ, respectively (1). Ψ increases the potential for an extra hydrogen bond between bases, compared to uridine, and contributes to structural stability and increased stacking interactions of the RNA (2). The Ψ modification is catalyzed by pseudouridine synthase (PUS), which either acts independently, or is bound to the guide RNA by a non-continuous bipartite 10–12 nt sequence complementarity to the target site (3). Recent studies using genome-wide mapping of Ψs (Ψ-seq) showed that hundreds of Ψs are induced on mRNAs, mostly by soluble PUS enzymes (4–7).

Whereas the modifications on rRNAs are performed in the nucleolus, guiding of modifications on spliceosomal U1, U2, U4 and U5 snRNAs as well as snRNP assembly takes place in the Cajal bodies (CB). These modifications are guided by small Cajal-body RNA species (scaRNA) that are bound by WDR79 protein (8). snRNA modifications were thought to be constitutive, but recent studies revealed that pseudouridylation can be induced by environmental signals at novel sites (9). One such example are the two Ψs that are induced under stress on U2 snRNA, compromising splicing (9,10).

*Trypanosoma brucei* is an important parasite that cycles between two hosts; such cycling requires major adaptation to changes in temperature and nutrient levels (11). Trypanosomes are known to harbor unique RNA processing pathways, such as *trans-*splicing (12) and RNA editing (13). In *trans-*splicing, the small RNA known as spliced leader RNA (SL RNA) donates a 5′ exon to all mRNAs (12).

Trypanosomes possess a rich repertoire of snoRNAs, larger than that of yeast despite a similar genome size. Trypanosome H/ACA-like RNAs are unique as they are composed of only a single hairpin compared to double hairpin in most other eukaryotes. Trypanosome H/ACA RNAs possess an AGA instead of an ACA box (14–19). Such single hairpin RNAs also exist in Archaea (20). Ψ-seq on rRNAs isolated from both life cycle stages of the parasite, the procyclic form (PCF) and bloodstream form (BSF), identified 68 Ψ, including 21 hypermodified sites in BSF, suggesting that pseudouridylation is developmentally regulated. Overexpression of snoRNA guiding hypermodified modifications enables better growth at elevated temperatures, contributing to the adaptation of the parasite while cycling between the two hosts (14).

In this study, we performed the first high-throughput mapping of Ψs on small RNAs in *T. brucei*. The study identified 42 Ψs on spliceosomal snRNAs that are mostly guided by snoRNAs. The Ψs are located in both RNA–RNA and RNA–protein interaction domains. The *T. brucei* snoRNAs bind the methyltransferase-associated protein (MTAP), the homologue of human WDR79 (21). *MTAP* silencing predominantly eliminated the Ψs on snRNAs and not rRNAs. The presence of only 83 H/ACA snoRNAs in *T. brucei* guiding at least 110 Ψs on spliceosomal snRNAs and rRNAs necessitates dual functionality; thus, several molecules must guide at least two targets. Indeed, *in vivo* UV induced cross-linking to U2 snRNA identified snoRNA species that are implicated in rRNA modification. Depletion of such guiding snoRNA compromised the snRNA modification and parasite growth. Finally, we provide evidence that Ψ strengthens RNA–RNA and RNA–protein interactions at elevated temperatures that are likely to be essential for parasite cycling between the hosts.

## MATERIALS AND METHODS

### Cell growth and transfection

Procyclic form (PCF) *T. brucei*, strain 29-13, which carries integrated genes for the T7 polymerase and the tetracycline repressor, was grown in SDM-79 medium supplemented with 10% fetal calf serum, in the presence of 50 μg/ml hygromycin and 15 μg/ml G418. The bloodstream form (BSF) of *T. brucei* 427 (cell line 131–514) was aerobically cultivated at 37°C under 5% CO_2_ in HMI-9 medium supplemented with 10% fetal calf serum, 2 μg/ml G418 and 2.4 μg/ml phleomycin (14).

### Construct preparation

Stem-loop constructs were generated to silence MTAP and snoRNA, using primers listed in Supplementary Table ST1, as described (14,17). snoRNAi (snoRNA interference) for TB11Cs6H1 was established using the Gateway recombination cloning system with minor modifications (22). Initially, mature snoRNA was cloned in pGEM-T easy vector (Promega) using the primers described in Supplementary Table ST1. Upon confirming the insert sequence, the snoRNA-pGEM vector was used as template for a polymerase chain reaction (PCR) using the primers Forward-AAATCTAGAGACGGCCAGTGAATTGTAA, Reverse –ATAACGCGTCCATGATTACGCCAAGCTAT. This PCR product was later cloned into the pCR 8/GW/TOPO vector (Invitrogen), and subjected to LR-recombination with the pTrypRNAiGate vector, resulting in a snoRNA stem–loop construct. Stem-loop constructs were linearized by EcorV digestion.

### (*N*-Cyclohexyl-*N*-β-(4-methylmorpholinium) treatment

RNA was treated with (*N*-cyclohexyl-*N*-β-(4-methylmorpholinium) (CMC) in bicine buffer (0.17 M CMC in 50 mM bicine, pH 8.3, 4 mM EDTA, 7 M urea) at 37°C for 20 min. Excess CMC was removed by ethanol precipitation. To remove CMC groups attached to G and U, the CMC-treated RNA was subjected to alkali hydrolysis with Na_2_CO_3_ (50 mM, pH 10. 4) at 37°C for 4 h, as previously described (23). The reacted RNA was recovered by phenol: chloroform extraction, and ethanol precipitation.

### Primer extension and Northern analyses

Primer extension was performed as previously described (17,24). The extension products were analyzed on 12% denaturing acrylamide gels. For northern analysis, total RNA was extracted, separated on either 10% acrylamide denaturing gel or agarose-formaldehyde gel, and analyzed using RNA probes. RNA probes were prepared by *in-vitro* transcription using α-^32^P-UTP. Primers used for *in-vitro* transcription are listed in Supplementary Table ST1.

### Preparation of small RNome

**Figure 1.**
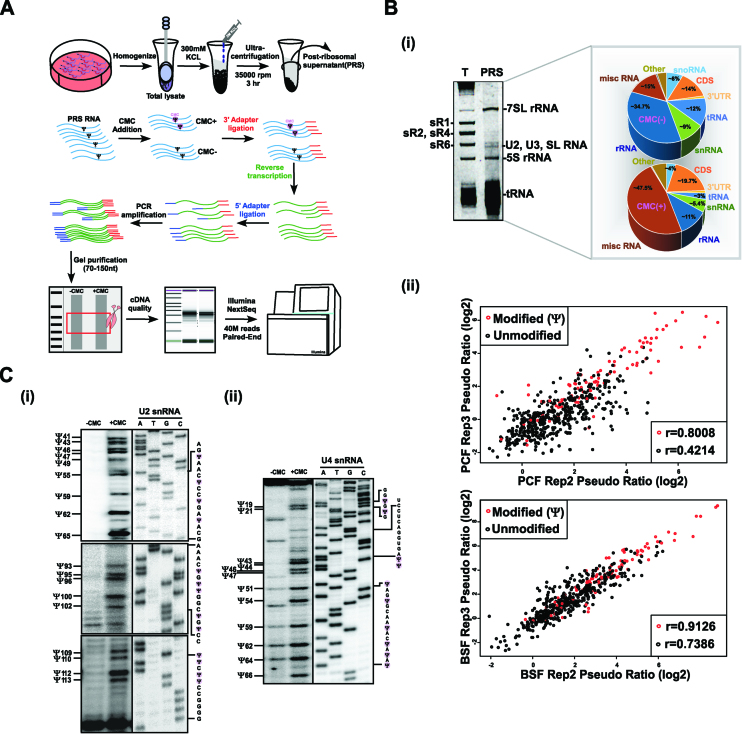
Small RNA Ψ-seq in *T. brucei*. (**A**) Schematic presentation of the small RNA Ψ-seq protocol. The scheme illustrates the extract preparation, fractionation, and the small RNA Ψ-seq methodology. (**B**) Enrichment of Ψs in small RNA Ψ-seq. Whole-cell extracts from 2 × 10^9^ PCF and BSF cells was prepared and depleted of ribosomes; RNA (800ng) was subjected to CMC treatment and used to prepare small RNA libraries, as described in (A). (i) Enrichment of small RNAs in PRS. RNA was separated on a 10% denaturing gel and stained with ethidium bromide. (T) total RNA; PRS. The identity of each RNA type is indicated. The pie diagram represents the types of RNA present in the Ψ-seq library. (ii) The reproducibility of pseudouridines in Ψ-seq libraries. Pairwise comparison of Ψ-fc(log2) from two libraries showing pseudouridylated sites (in red) and non-modified sites (in black) in snRNA. Pearson's correlation coefficient (r) is indicated. Fourteen independent biological replicates were used to detect Ψs in small RNA Ψ-seq. (**C**) Validation of Ψs in T. *brucei* U2 and U4 snRNA. Total RNA (100μg) treated with CMC (+CMC) or untreated (–CMC) was subjected to primer extension with region specific primers and analyzed on a 12% polyacrylamide gel (7M urea). The results along with DNA sequencing performed using the same primer are presented for U2 (i) and U4 (ii) snRNA. The position of the Ψs are indicated (one nt after the actual stop seen in the gel), as well as the RNA sequence. Contrast adjusted blots are separated by bold lines.

Whole cell extracts were prepared from 10^9^ cells; after extraction with 0.3M KCl, the ribosomes were removed by centrifugation for 3 h at 35 000 rpm in a Beckman 70.1Ti rotor (150 000 × g) (Figure 1A). RNA extracted from the post-ribosomal supernatant (PRS) was used for library preparation, essentially as described (14).

### Small RNA Ψ-seq and detection of pseudouridylated sites

To perform small RNA Ψ-seq, an adaptor was ligated to the 3′ end of the PRS RNA (without fragmentation) or total RNA (upon fragmentation) before and after CMC treatment, and cDNA was prepared using AffinityScript reverse transcriptase (Agilent). The cDNA was then ligated to an adaptor, PCR amplified, and after size selection on E-Gel EX (Invitrogen), the samples were sequenced in an Illumina NextSeq machine in paired end mode (40 million reads for each sample).

The 42 bp sequence reads obtained from the Illumina Genome Analyzer were first trimmed of Illumina adapters using the FASTX toolkit (http://hannonlab.cshl.edu/fastx_toolkit), and reads of 15 bases or less were discarded from subsequent analysis. The remaining reads were mapped to the *T. brucei* genome (TriTrypDB-2.5 http://tritrypdb.org/common/downloads/release/) using SMALT v0.7.5 (http://www.sanger.ac.uk/resources/software/smalt/) with the default parameters. Only properly paired partners were retained. Each read pair was ‘virtually’ extended to cover the area from the beginning of the first read to the end of its partner. For each base, the number of reads initializing at that location as well as the number of reads covering the position were calculated. A combination of Bedtools (http://bedtools.readthedocs.io/en/latest/) and in-house Perl scripts was used to calculate the Ψ-ratio and Ψ-fc (fold change), as previously described.

For each nucleotide, we computed the Ψ-ratio, dividing the number of reads covering that nucleotide by the number of nucleotides initiating at the following base (i.e. corresponding to the last position copied by the reverse transcriptase). This was performed for both (–CMC) and (+CMC) samples. The Ψ-fc(log_2_) was computed as the log_2_-fold change of the Ψ-ratios in (–CMC) versus the (+CMC) samples (4). Applying a strict threshold, the putative Ψ sites were called based on the following criteria: a Ψ-fc (log_2_) of 1.15 or greater (equivalent to >2.2-fold change), a Ψ-ratio of CMC treated sample >0.01, and with a minimum of 800 reads initiating at the (*n* + 1) nucleotide. We applied this threshold to each PCF and BSF sample. Our small RNA Ψ-seq protocol calls a uridine as Ψ only if it was detected in at least five independent biological replicates, and was also reduced under *CBF5* silencing experiments. We then merged the positions to generate a list of all positions meeting the criteria (Supplementary Figure S1). For each developmental stage (PCF and BSF), we calculated the Ψ-ratio and Ψ-fc for all called Ψ sites. Hypermodified sites were identified if Ψ-fc(log_2_) (BSF/PCF) >1.3 (equivalent to ∼2.5-fold increase) in at least two independent biological replicates (Supplementary Figure S3).

### PERL and SHELL scripts

The scripts used to analyse small RNA Ψ-seq is provided in the Supplementary Files. ‘Run_Pipeline.sh’ is a shell script wrapper for the pipeline running small RNA Ψ-seq. The Ψ-detection algorithm receives as input two sets of paired-end fastq files for the –CMC and +CMC conditions accordingly. It then runs the SMALT alignment program against the spliceosomal snRNA or rRNA database to produce SAM files which are then converted to BAM files using samtools. The BAM files are then converted to bed files where only properly paired reads are further analyzed. The bed files are further used as input to the genomeCoverageBed program from the bedtools suite (v2.26.0). In addition, a short perl script ‘Count_Initiating.pl’ tabulates the summation of the reads terminating at each base. The perl script ‘ModCount_w_initiating _general_bp.pl’ is then called using all these files as input to output a detailed account of the Ψ fold-change (Ψ-fc) at each base.

### Quantification of pseudouridine level by LC-MS

RNA (300 ng) was digested using 4U of P1 nuclease (Sigma N8630) in 25 mM NaCl 2.5 mM ZnCl_2_ for 2 h at 37°C, followed by the addition of 5 units of Antarctic Phosphatase (New England Biolabs). The mixture was incubated at 37°C for another 2 h. The reaction was analyzed using LC–MS/MS. The nucleosides were separated by ultra-performance liquid chromatography (Vanquish UHPLC Thermo Fisher scientific) on a Hyprsil GOLD aQ column (Thermo Scientific 25303-152130), and then detected by Q Exactive™ HF hybrid quadrupole-Orbitrap mass spectrometer (Thermo Scientific) in Parallel Reaction Monitoring Mode (PRM). The mass transitions of *m*/*z* 243.06 to 153.03, 183.04 (pseudouridine), *m*/*z* 243.06 to 110.02, 82.02 (U) in the negative mode, and mass transitions of *m*/*z* 268.10 to 136.06, (A), m/z 284.1 to 152.06 (G) and m/z 244.1 to 112.05 (C) in the positive mode were monitored and recorded. Concentrations of nucleosides in RNA samples were deduced by fitting the signal intensities using standard curves.

### FPLC sizing column fractionation of RNPs

Whole cell extracts from 2 × 10^9^ cells were prepared and loaded on a Superdex 200 gel filtration column (Amersham BioSciences), equilibrated with 20 mM HEPES (pH 7.9), 10 mM MgCl_2_, 150 mM KCl, at a flow rate of 0.5 ml/min. Fractions of 0.5 ml were collected. RNA and protein from every second fraction were analyzed by western and northern blotting. The elutions of bovine serum albumin (66 kDa) and β-amylase (200 kDa) were used as markers to follow the fractionation (19).

### Generation of antibodies to MTAP

The C/N-terminal sequence of MTAP (Tb927.11.16490) was cloned into the pET28 vector. Primers are listed (Supplementary Table ST1). Recombinant protein was purified using the Bug-buster reagent (Novagen, Inc.), and 400 μg of purified protein was used for multiple injections into rabbits.

### *In vivo* cross-linking with AMT-psoralen and affinity selection

Cross-linking was performed essentially as described in (25). Briefly, *T. brucei* cells were harvested and resuspended at 5 × 10^7^ cells/ml, and washed twice with PBS. Cells (∼10^9^) were concentrated and incubated on ice. 4′-Aminomethyl-trioxsalen hydrochloride (AMT) (Sigma) was added to the cells at a concentration of 0.2 mg/ml. Cells treated with AMT were kept on ice and irradiated using a UV lamp at 365 nm at a light intensity of 10 mW/cm^2^ for 30 min. Next, the cells were washed once with PBS and deproteinized by digestion with proteinase K (Roche) (200 μg/ml in 1% SDS for 60 min). RNA was prepared using TRIzol (Sigma) reagent. Approximately 250 μg of RNA was used for affinity selection, essentially as described (25), using anti-sense U2 snRNA oligonucleotide. After affinity selection, the RNA was used to prepare small RNA libraries, as described (14).

### Generation of the small RNA interactome

*T. brucei* PCF cells (2 × 10^9^) were incubated with AMT-psoralen, and extracts and PRS were prepared as described above. RNA was prepared from the PRS and was subjected to mild fragmentation in Tris buffer (10 mM Tris–HCl (pH 8.0 at 37°C), 5 mM MgCl_2_, 0.1 M KCl, 0.02% Triton X-100 and 0.1 mg/ml BSA) by boiling at 94°C for 3 min. The RNA was dephosphorylated using alkaline phosphatase and purified on SPRI-beads (Agencourt^®^ AMPure^®^ XP). The RNA was then ligated using RNA ligase (Thermo Scientific) at 25°C overnight, and again purified; cross-linking was reversed by irradiation at 254 nm. The recovered RNA was then used for library preparation, as previously described (14).

### Native gel for detecting the U4/U6 dimeric complex

Total RNA (∼300 μg) from *T. brucei* PCF before or after 2.5 days of *CBF5* silencing (24) was suspended in 10 mM HEPES pH 7.5, 100 mM KCl, boiled for 2 min, and slow-cooled to room temperature. The RNA (30 μg) was aliquoted and incubated with 28 μM U4 and U6 oligonucleotides at room temperature for 5 min. The annealed RNA was incubated at the different temperatures for 15 minutes and placed on ice. The RNA was loaded on a native 12% polyacrylamide gel (prepared from a stock of 29:1 acrylamide-bis acrylamide) in Tris–glycine buffer, pH 8.3. The gel was subjected to Northern analysis after treatment with 8.3 M urea, and probed with either U4 or U6 anti-sense RNA probes.

### Microfluidic device fabrication

Microfluidic devices were fabricated as previously described from polydimethylsiloxane (PDMS) (SYLGARD 184, Dow Corning, USA) (26). Briefly, two layers of PDMS were aligned to create an integrated device that was designed using AutoCAD1. Molds were fabricated using soft lithography and the device included 32 independent channels, each with 16 MITOMI valves to maintain any interaction beneath it at equilibrium. The device could simultaneously test 32 concentrations.

### U2B'' and U2A’ protein expression and immobilization

The template for N' terminal cMYC, and C' terminal 6 x HIS U2B" (Tb927.3.3480) or U2A’ (Tb927.10.14360) synthetic gene was created by assembly PCR (26) using primers listed in Supplementary Table ST1. U2B" and U2A’ protein containing 5′-cMyc and 3′-HIS tag was synthesized using rabbit reticulocyte quick coupled transcription and translation system (Promega). The 5′-cMyc and 3′-HIS tagged U2B" and U2A’ protein was then immobilized underneath the MITOMI valve using anti HIS-biotin (33 μg/ml, QIAGEN) and detected using anti cMyc-Cy3 (13 μg/ml, Sigma-Aldrich) as previously described (26). The fluorescent signal intensities were measured using a microarray laser scanner (LS Reloaded, TECAN) and the data were extracted using GenepixPro software.

### Data analysis—*K*_d_ determination

Binding affinity was determined by normalizing the Cy5 tagged RNA signal to the protein signal. The dose response relative binding was then fitted to the following equation using non-linear least squares minimization (http://statpages.org/nonlin.html): ${K_{\rm d}} = \frac{{{{[ {\rm A} ]}^x} + \ {{[ {\rm B} ]}^y}}}{{[ {{{\rm A}_x}{{\rm B}_y}} ]}}$, wherein [A]^*x*^, [B]^*y*^ and [A_*x*_B_*y*_] are the equilibrium concentration of A, B and AB respectively.

## RESULTS

### Mapping Ψs on *T. brucei* snRNAs by small RNA Ψ-seq

The finding that *T. brucei* has more Ψs on its rRNAs than yeast led us to map the Ψs on spliceosomal snRNAs. We sought to establish a novel protocol for genome-wide mapping of Ψs on small RNAs. To enrich for small RNAs, whole-cell extracts were prepared from both *T. brucei* PCF and BSF parasites and were depleted of ribosomes, resulting in a post-ribosomal supernatant (PRS) enriched in small RNAs (Figure 1A). For Ψ mapping, RNA was prepared from PRS and treated with *N*-cyclohexyl-*N*-β-(4-methylmorpholinium) (CMC) that selectively binds to Ψ (23). Small RNA Ψ-seq libraries were prepared, and as shown by the pie diagram (Figure 1Bi) the libraries were enriched for small RNAs. This protocol succeeded in reducing the level of rRNAs from 80% to <40%. To locate the Ψs, we used a pipeline that determines the ratio of the number of reads supporting reverse transcriptase (RT) termination versus the number of read-throughs (known as the Ψ-ratio) (4). All experiments were compared with samples sequenced in the same run. Ψs detected from 14 biological small RNA Ψ-seq replicates and primer extension mapping are presented in Supplementary Figure S1. Our small RNA Ψ-seq protocol considers a uridine as Ψ only if it was detected in at least five libraries, was reduced upon *CBF5* depletion (see below), and the scatterplots show a high correlation between the experiments (*r* = 0.9 for modified positions) (Figure 1Bii). Our analysis identified 42 Ψs on spliceosomal snRNAs in at least five independent libraries, and 31 Ψs in 10 or more independent libraries. The difference in number of Ψs detected in each library depends on the amount of snRNA present in different PRS used for library preparation that can vary because of minor variations in salt concentration. Only if each of the transcript is represented by numerous reads it is possible to detect those Ψ that are less abundant, suggesting that the level of Ψ differs along the RNA. Thus, many independent biological replicates are necessary to detect and quantify individual Ψs using small RNA Ψ-seq. In addition, primer extension on *T. brucei* U2 and U4 snRNA verified the positions suggested by small RNA Ψ-seq (Figure 1C). However, those sites that were not detected by all libraries are also likely to exist. For example, primer extension verified the existence of Ψs such as Ψ47 and 100 in U2 snRNA (Figure 1Ci); Ψ47, 51, 54, 64 and 66 in U4 snRNA (Figure 1Cii) and Ψ33 and 46 in U6 snRNA (Supplementary Figure S2C) which were detected in <10 small RNA Ψ-seq libraries (but more than five libraries), suggesting that even if a modification exists in only a fraction of the libraries, it is a valid modification.

Our small RNA Ψ-seq also detected Ψs on other small RNA species such U3, C/D and H/ACA snoRNA, 7SL RNA, and also verified the presence of Ψ on SL RNA (16), and these results will be presented elsewhere. In this study, we focused on the Ψs on spliceosomal snRNAs involved in splicing and its functional implications.

**Figure 2.**
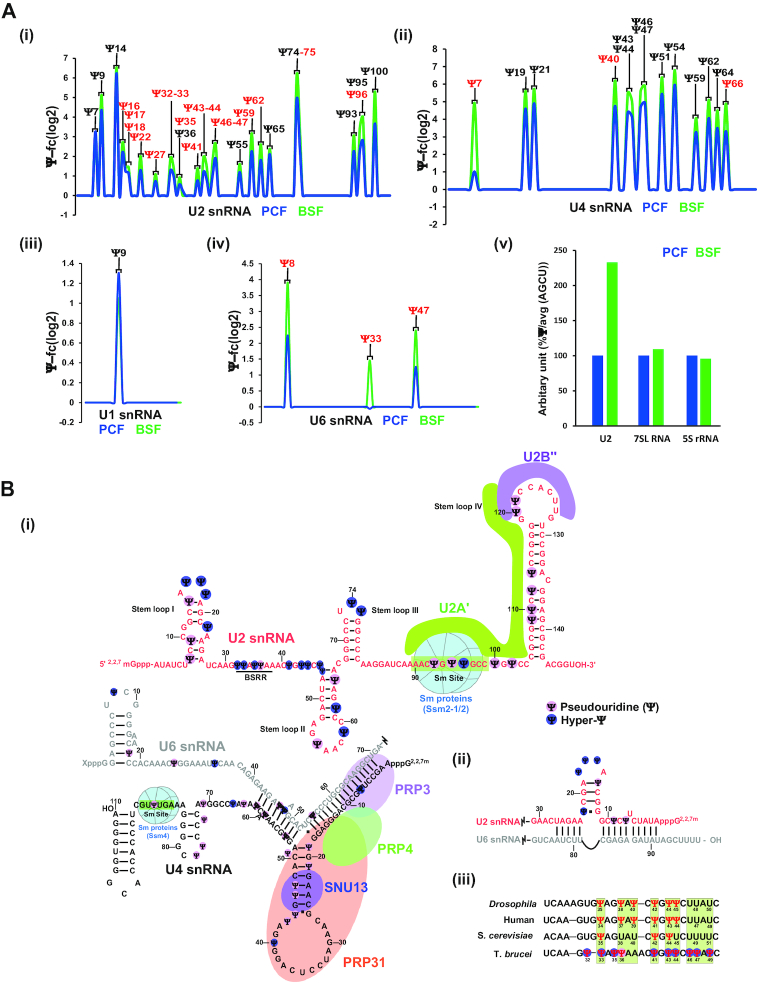
Genome wide small RNA Ψ-seq. (**A**) The position of Ψs is indicated based on small RNA Ψ-seq. A reprentative line-graph of the Ψ-fc(log_2_) is presented for U2 snRNA (i), U4 snRNA (ii), U1 snRNA (iii) and U6 snRNA (iv) from PCF and BSF RNAs. Hypermodified Ψs are highlighted in red. Fourteen independent biological replicates were used to detect Ψs in small RNA Ψ-seq. Three independent replicates were used to identify hypermodified Ψs. PCF and BSF Ψs were compared from the same small RNA Ψ-seq sequencing experiment. (v) Hypermodification in U2 snRNA was validated by mass spectrometry. (**B**) Ψs are enriched in RNA–RNA and RNA-protein interaction domains of snRNAs. Scheme depicting the position of *T. brucei* Ψs on the secondary structure of snRNAs based on small RNA Ψ-seq and primer extension, highlighting functional domains (BSRR, Sm site, and protein and RNA interaction regions). The hypermodified positions (BSF/PCF fold change > 2.5 (log_2_FC>1.3)) present in BSF RNA are marked (dark blue). (i) U2, U4 and U6 interaction, (ii) U2/U6 interaction, (iii) comparison of Ψs in BSRR among different species. The data were derived from (48).

The quality of PCF and BSF small RNA Ψ-seq was validated by the detection of a single Ψ28 on SL RNA and Ψ74 on 5.8S rRNA that remained invariant between the two life stages (Supplementary Figure S2A). It was previously demonstrated that Ψ-seq can detect relative stoichiometry among Ψs (4,5). Accordingly, the modifications on spliceosomal snRNAs were determined in both PCF and BSF RNA (Figure 2A and Supplementary Figure S3), showing among 27 Ψ sites on U2 snRNA in both life stages of the parasite; 17 positions were hypermodified on U2 snRNA derived from BSF (i.e. Ψ-fc of BSF/PCF > 2.5). This striking observation was verified by mass spectrometry analysis (Figure 2Aiii). Comparison of our data presented in this study, to published analyses of yeast and human suggest that *T. brucei* possess the highest number of Ψs (61 Ψ) reported thus far on these RNA types. *T. brucei* U1 snRNA has 4 Ψs compared to 2 Ψs in human and 2 Ψs in yeast. Similarly, *T. brucei* U2, U4, U5 and U6 snRNA has 34, 14, 2 and 7 Ψs respectively compared to 14, 3, 4 and 3 Ψs in human, whereas yeast has only 5 Ψs in U2, and 1 Ψs in U5 snRNA.

The location of Ψs on secondary structure of *T. brucei* spliceosomal snRNAs is depicted in Figure 2B and Supplementary Figure S2B, demonstrating the presence of Ψs not only in RNA–RNA interaction domains but also in protein binding domains. Ψs were also detected in the U2 domain implicated in the interaction with the pre-mRNA (branch site recognition region; BSRR) (Figure 2Biii). Previously, it was suggested that in contrast to yeast, the branch point sequence in trypanosomes is not conserved in a manner that would suggest extensive base-pairing between U2 and pre-mRNA (27). The presence of four Ψs (Ψ32, 33, 35 and 36) in this domain may change our understanding of the pre-mRNA U2 base-pair interactions in trypanosomes (see Discussion). The Ψs in *T. brucei* BSRR is compared to homologous domains in other organisms, showing conservation in this functional domain (Figure 2Biii).

### All trypanosome spliceosomal snRNA Ψs are guided by snoRNAs

**Figure 3.**
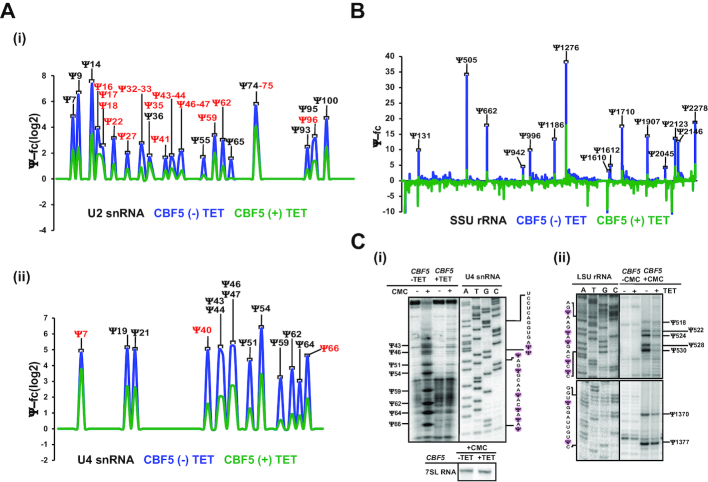
*T. brucei* snRNA Ψs are guided by H/ACA snoRNA. (**A**) *CBF5* silencing affects snRNA Ψs. Ψ-fc(log2) values (y-axis) were determined for both *CBF5* -TET and +TET based on small RNA Ψ-seq libraries. Representative line graphs of U2 (i) and U4 snRNA (ii) are presented. Ψs hypermodified in BSF are shown in red. Three independent biological replicates of small RNA Ψ-seq was used to validate the dependence of *T. brucei* snRNA Ψs upon *CBF5* silencing. (**B**) The effect of *CBF5* silencing on rRNA Ψ. Ψ-fc values (y-axis) were determined for both *CBF5* –TET and +TET based on Ψ-seq libraries. Representative line graphs of SSU rRNA are presented. (**C**) Validation of the effect of *CBF5* silencing on snRNA and rRNA Ψs. Total RNA from cells carrying the *CBF5* silencing construct, either uninduced (−TET) or induced (+TET) was subjected to CMC treatment and analyzed by primer extension next to the DNA sequence, using the same primer reaction. The Ψ positions and the RNA sequence are indicated. Contrast adjusted blots are separated by bold lines.

To determine whether indeed most *T. brucei* spliceosomal snRNA Ψs are guided by snoRNAs, mapping of the Ψ was performed by small RNA Ψ-seq on cells depleted for *CBF5* (H/ACA snoRNA associated pseudouridine synthase) (24). All detected Ψs on the spliceosomal snRNAs were diminished in the *CBF5*-silenced cells (Figure 3A), indicating that as in metazoa and in contrast to yeast (28), the H/ACA snoRNAs direct the Ψs on *T. brucei* snRNAs. The reduction in the level of modifications of the different sites on the U snRNAs (based on three independent biological replicates) was highly significant (*P* < 0.001) (Supplementary Figure S4A-D). This result was also validated by mass spectrometry (Supplementary Figure S4B). Interestingly, modification was reduced to various extents for the different sites, and was correlated with differential reduction in the level of snoRNAs (24). The reduction in the level of Ψs on U2 and U4 spliceosomal snRNAs (Figure 3A), and on rRNAs (Figure 3B) upon *CBF5* silencing is presented, as is the reduction of the modifications on SL RNA, 5.8S rRNA, U1, and U6 snRNAs (Supplementary Figure S4C). The small RNA Ψ-seq upon CBF5 silencing was also confirmed by primer extension on U4 snRNA (Figure 3C).

### MTAP binding to H/ACA snoRNA is essential for its ability to guide modifications on spliceosomal snRNAs

**Figure 4.**
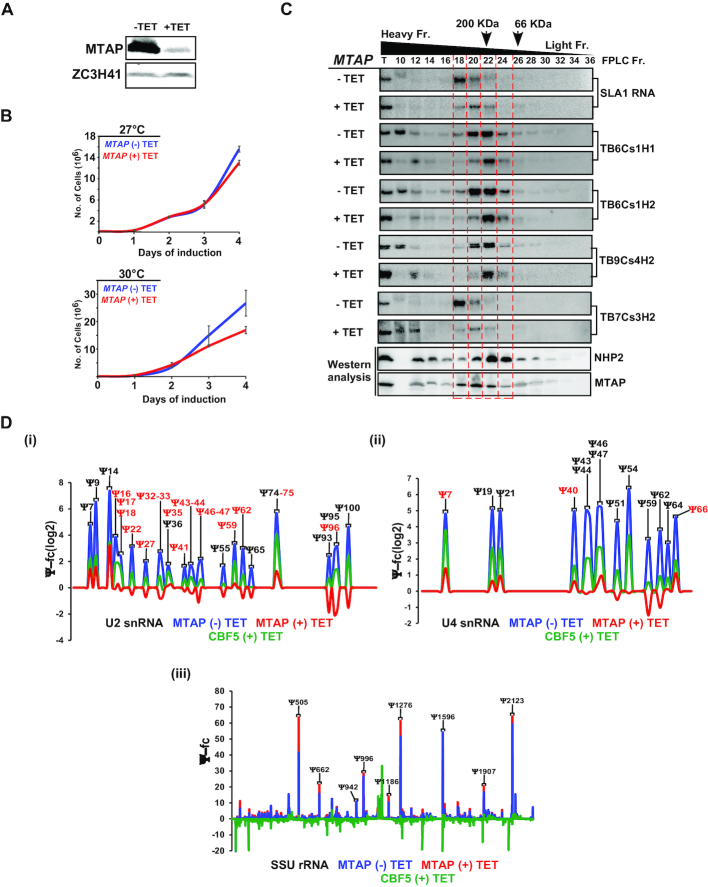
Ψs on *T. brucei* snRNAs depend on MTAP. (**A**) *MTAP* silencing. (i) Cells carrying the silencing construct for MTAP were silenced for 2.5 days, and protein from (3 × 10^7^) cells was subjected to Western analysis with MTAP antibodies (diluted 1:10 000). (**B**) Growth of cells upon *MTAP* silencing. Uninduced cells carrying the silencing construct (-TET) were compared with cells induced for silencing (+TET) at 27°C (upper panel) and 30°C (lower panel). Data are presented as mean ± S.E.M. Experiments were done in triplicate (*n* = 3). (**C**) snoRNPs are present in two distinct RNP complexes. Whole cell extracts from 10^9^ uninduced (–TET) or induced (+TET) cells carrying the *MTAP* silencing construct were fractionated on a FPLC Superdex column. Fractions were deproteinized, and RNA was subjected to Northern blot analysis with the indicated RNA probes. Fractions were also subjected to Western analysis. The positions of marker proteins BSA (66KDa) and β amylase (200kDa) in the fractionation are indicated by arrows. The heavy and light fractions are indicated. (**D**) *MTAP* silencing abolishes the Ψs only on snRNAs. Ψ-fc(log2) values (y-axis) were determined for *MTAP*-TET, *MTAP* +TET and *CBF5* +TET based on small RNA Ψ-seq libraries sequenced in the same experiment. Representative line graphs of U2 (i), U4 snRNA (ii) and SSU rRNA (iii). Ψs hypermodified in the BSF stage are shown in red. Four independent biological replicates of small RNA Ψ-seq was used to validate the dependence of *T. brucei* snRNA Ψs upon *MTAP* silencing. Ψs from *CBF5* and *MTAP* silencing were compared from the same small RNA Ψ-seq sequencing experiment.

*T. brucei* MTAP was disovered as the protein that assoicates with the complex that governs the methylation of SL RNA (21). The *T. brucei* MTAP is homolgous to human WDR79, that binds scaRNAs in Cajal bodies (8). Here, we examined the possibility that MTAP binds to trypanosome H/ACA snoRNAs as in humans (8). To this end, *MTAP* was silenced and its silencing was confirmed (Figure 4A). Silencing of *MTAP* resulted in a growth defect at elevated temperature (Figure 4B). Next, the association of MTAP with snoRNAs was examined, before and after MTAP silencing. Whole cell extracts were fractionated on a FPLC S-200 Superdex column, and the extracted RNA was subjected to Northern analysis. A shift in the hybridization pattern of snoRNP was observed upon *MTAP* silencing; the heavier RNPs disappeared, while the smaller RNP population was retained (Figure 4C, Supplementary Figure S5), suggesting that under normal conditions, two types of snoRNP populations exist; one snoRNP bound to MTAP (and its associated proteins) and core proteins, and another snoRNP that is bound only to the core proteins.

Next, we mapped Ψs using small RNA Ψ-seq following *MTAP* silencing (Figure 4Di–ii). Indeed, marked reduction in Ψs were observed only on the spliceosomal snRNAs but not the rRNA pool (Figure 4Dii). Results obtained (four independent biological replicates) indicate significant (*P* < 0.001) and major reductions in spliceosomal snRNA modifications, but no reduction in rRNA modification (Supplementary Figure S6A). The results of small RNA Ψ-seq following MTAP silencing were also confirmed by primer extension on rRNA and U4 snRNA (Supplementary Figure S6B, C). Note that the silencing of MTAP affected growth at elevated temperatures (Figure 4B), further suggesting that MTAP is essential in these parasites mostly for spliceosomal snRNAs but not for rRNA modification.

### *In vivo* AMT-cross linking identifies the snoRNAs that interact with U2 snRNA

**Figure 5.**
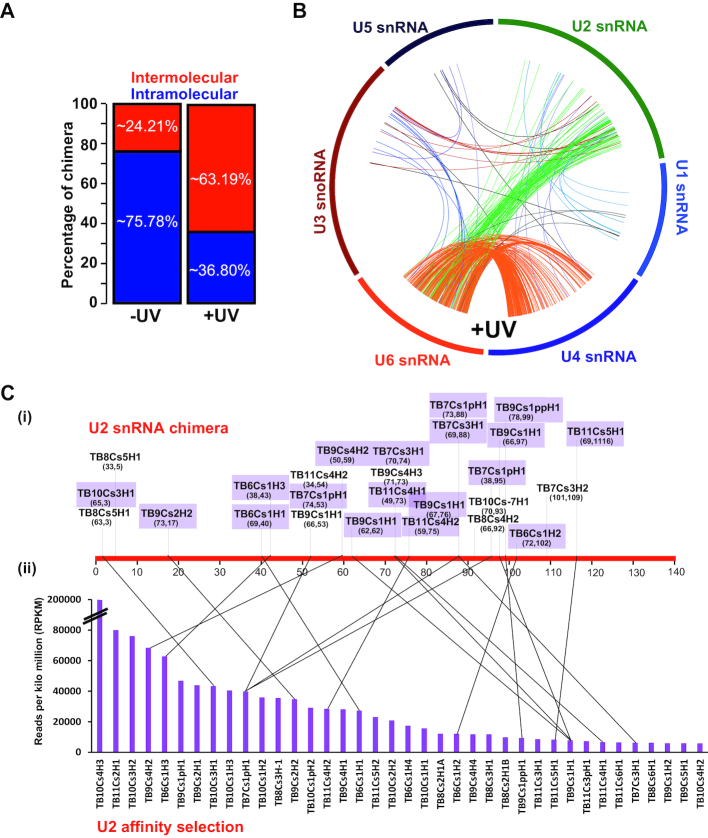
*T. brucei* small RNA interactome. (**A**) *In vivo* AMT-psoralen UV cross-linking was performed and used to identify interacting RNA molecules upon ligation. The RNA (-UV) and (+UV) was analyzed for intermolecular or intramolecular cross-links. The percentage of each of these species is presented. (**B**) Circos plot representing the interactome of snRNAs. The reads for these interactions are shown in (Supplementary Figure S8). (**C**) Validation of chimera between U2 snRNA and snoRNAs. (i) The ligation points of sn(o)RNA to U2 snRNA along its sequence following UV treatment are depicted on a linear scale; the control (-UV) is presented in (Supplementary Figure S9). Coordinates of the obtained chimera are given as the nucleotide on snoRNAs ligated to the position on U2 snRNA. (ii) Identification of snoRNAs that were affinity selected from cells subjected to AMT cross-linking, and affinity selection with U2 snRNA anti-sense oligonucleotide. The RPKMs of the different snoRNAs are presented as a bar diagram. The lines specify snoRNAs that were detected in both experiments used to identify the snoRNAs interacting with U2 snRNA.

The presence of 34 Ψs on U2 snRNA is intriguing and prompted us to identify the H/ACA snoRNAs that guide these modifications. To this end, the small RNA interactome of *T. brucei* was determined using *in vivo* cross-linking, similar to the recently described methodology (29). Indeed, UV cross-linking enriched for intermolecular interactions such as U4/U6 and U6/U2 (Figure 5A, Supplementary Figures S7–S8), as shown in Figure 5B. Enrichment of the U2/U6 and U4/U6 interactions suggests that the cells used were metabolically active (30). The snoRNAs that were ligated to U2 snRNA were compared between the (−)UV and (+)UV samples (Supplementary Figure S9), and the list is presented in Figure 5Ci. Twenty-four snoRNAs were found in the (+)UV sample, and only 11 in the (−)UV control (Supplementary Figure S9). Our small RNA interactome also detected several novel interactions between *T. brucei* C/D snoRNA–snRNA and other small RNA species, but here we present only the data relevant to spliceosomal snRNAs.

To obtain an independent confirmation of the specific cross linking of snoRNA and U2 snRNA, total RNA was extracted from the AMT-psoralen treated cells following UV irradiation, and was used to affinity select the RNA interacting with U2 snRNA. The reads per kilo million (RPKM) of the snoRNA cross-linked to U2 shows that only a subset of snoRNAs were efficiently selected with U2 snRNA (Figure 5C). A line connecting the upper (Figure 5Ci) and lower (Figure 5Cii) panels indicates that more than 50% the snoRNAs were enriched in both approaches. This experiment (Figure 5) identified the majority of snoRNAs that interact with U2 snRNA, presumably to guide the Ψs. The interaction domain between the snoRNAs and the target can be used to deduce the snoRNA that guides each specific site. To this end, we first inspected known interactions such as SLA1/SL RNA and H/ACA snoRNA with its rRNA targets (Supplementary Figure S8). The results suggest that the chimera was generated close to, but not with in the pseudouridylation pocket. This was also the case for the U2-snoRNA chimera (Supplementary Figure S9). Thus, the cross-linking approach can be used to identify the snoRNAs that guides the Ψs on U2 snRNA.

### Ablation of snoRNA guiding Ψ on spliceosomal snRNA affects growth and *trans*-splicing

**Figure 6.**
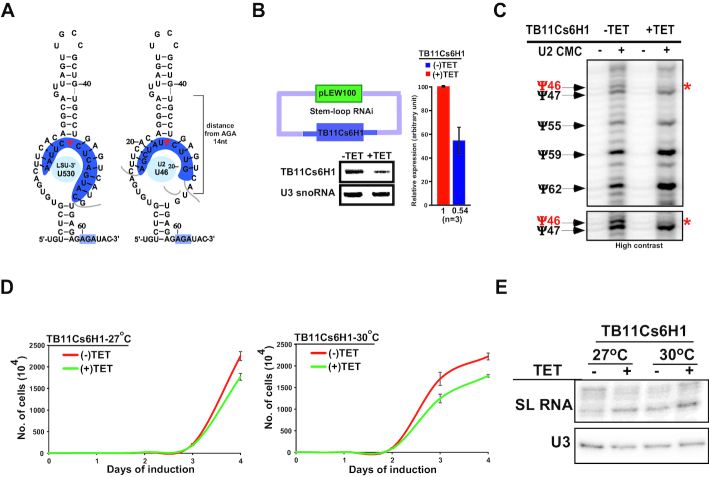
Single hairpin-snoRNA guide snRNA Ψ. (**A**) TB11Cs6H1 snoRNA can potentially guide Ψ on both rRNA (Ψ1377) and U2 snRNA (Ψ46). The potential secondary structure that can support pseudouridylation of the two different substrates is presented. (**B**) TB11Cs6H1 silencing. Cells expressing the snoRNAi silencing construct were induced for 4 days, and the RNA from uninduced and silenced cells was subjected to Northern analysis. A bar graph representing the silencing from three different experiments is presented. (**C**) TB11Cs6H1 snoRNA guides Ψ46 in U2 snRNA. RNA from uninduced (–TET) cells, or cells silenced for 4 days (+TET) was subjected to CMC mapping by primer extension. The Ψs are presented, and the effect on the predicted position is marked by a red asterisk. A contrast adjusted blot is shown in the lower panel. (**D**) Growth of cells upon TB11Cs6H1 snoRNA silencing. Uninduced cells carrying the snoRNA silencing construct (–TET) were compared with cells induced for silencing (+TET) at 27°C (upper panel) and 30°C (lower panel). Data are presented as mean ± S.E.M. Experiments were done in triplicate (*n* = 3). (**E**) Splicing defect upon TB11Cs6H1 snoRNA silencing. Total RNA (5μg) from uninduced (–TET) and induced (+TET) cells was subjected to primer extension and separated on a 6% polyacrylamide gel. U3 snoRNA served as a loading control.

The definitive proof for the snoRNA function in guiding a specific modification on snRNA may be achieved by eliminating the snoRNA and demonstrating an effect on the predicted modification. To this end, we chose TB11Cs6H1 H/ACA snoRNA which could potentially guide the hypermodified Ψ46 on U2 snRNA. The proposed interaction of this snoRNAs with its targets are illustrated (Figure 6A). The guiding rules for this snoRNA on rRNA Ψ530 are conventional, whereas the Ψ on snRNA is guided by the same pseudouridylation pocket but using more flexible rules. Note that the pocket guiding the Ψ46 carries two potential non-canonical base-pairs (Figure 6A). A cell line expressing the silencing construct of TB11Cs6H1 snoRNA was generated and the silencing led to reduction snoRNA (Figure 6B). A clear reduction was also observed in the predicted U2 Ψ46 upon silencing (Figure 6C). Next, we examined whether silencing of the snoRNA affected growth, especially at elevated temperature, since the Ψs guided by this snoRNA is hypermodified in BSF U2 snRNA. A clear growth defect at an elevated temperature was observed upon snoRNA silencing (Figure 6D). However, we can not exclude the likelihood that the growth defect could also arise from ablation of Ψ on rRNA or any other uncharacterized RNA target guided by the same snoRNA. A distinct effect on *trans*-splicing was also observed based on the accumulation of the SL RNA, as a result of its not being utilized in *trans*-splicing (Figure 6E).

### Implication of Ψs on RNA and protein interactions

**Figure 7.**
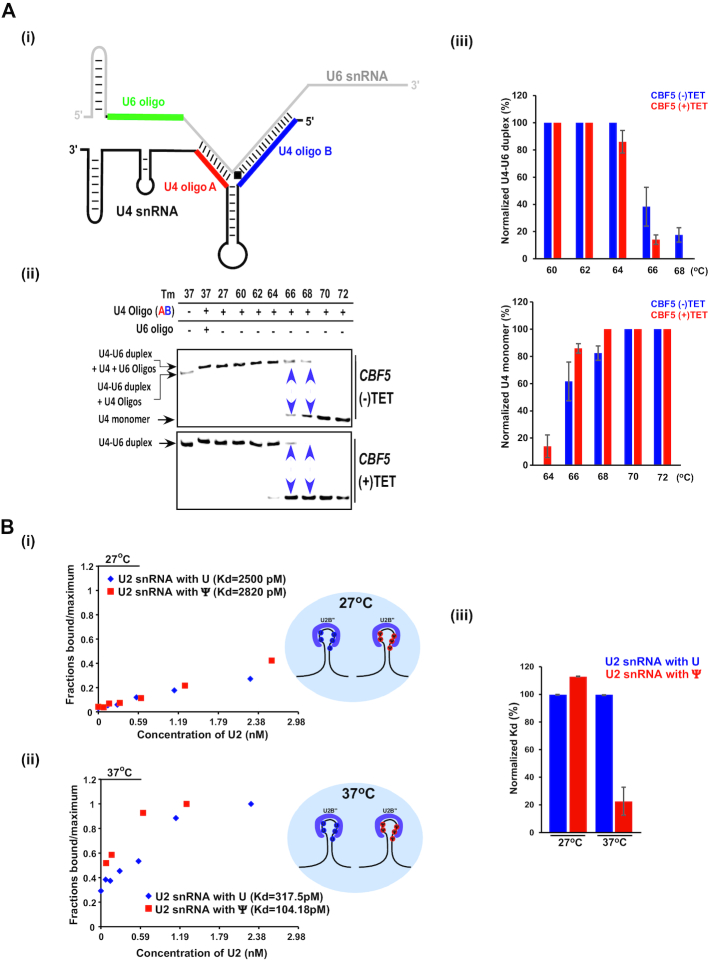
Ψs regulate RNA and protein interactions at elevated temperature. (**A**) The stability of the U4/U6 duplex depends on its Ψs. (i) Schematic representation showing the position of the oligonucleotides used for examining the dimeric U4/U6 complex. (ii) U4/U6 duplexes were reconstituted from total RNA derived from cells carrying a *CBF5* silencing construct, either uninduced (-TET) or after 2.5 days of silencing (+TET). Complexes were incubated with the indicated oligonucleotide, and incubated at different temperatures as indicated. Annealed RNA was separated on a 12% native gel and subjected to Northern analysis with a U4 RNA probe. The purple arrowheads represent the dissociation of the U4/U6 duplex. (iii) Percentage of U4/U6 duplex and U4 monomer was calculated from three independent biological replicates (as shown in Supplementary Figure S10B) using ImageJ software (https://imagej.nih.gov/ij/). Data are presented as mean ± S.E.M. (**B**) A dose response of Cy5 labeled U2 snRNA binding to U2B'' protein in a microfluidic device. After the U2B'' protein-U2 snRNA interaction reached equilibrium at either 27°C (i) or 37°C (ii), the free and U2B'' bound RNA concentration was measured. The data were normalized to maximum, and affinity was calculated by non-linear least squares fitting. Representative graph of three independent replicates is presented. (iii) Normalized *K*_d_ was calculated for the interaction of U2B" protein with U2 snRNA carrying either Ψ or U. Data are presented as mean ± S.E.M. Experiments were done in triplicate (*n* = 3).

Since the depletion of spliceosomal snRNA Ψs affected growth at elevated temperature (Figures 4B and 6D), we sought to identify interactions that might be heat-sensitive in spliceosomal snRNPs. Our data indicate that Ψs and hypermodified Ψs are present in domains involved in RNA/RNA-interactions, such as U2/U6 (Figure 2Bii), that take place during the splicing reaction, but also in U4/U6 interaction domains (Figure 2Bi) that bind within the dimeric particles before joining the active spliceosome (31). The many modifications on the U4/U6 interaction domain are likely to affect the stability of this duplex in trypanosomes. To examine whether the presence of Ψs enhances the stability of the U4/U6 interaction, RNA was prepared from un-induced cells (–TET) and cells induced for *CBF5* silencing (+TET). To assess the stability of the U4/U6 dimeric complex, total RNA was denatured and annealed with oligonucleotides A and B (shown in color in Figure 7Ai). These oligonucleotides base-pair with free RNA, preventing the self-annealing of the monomeric RNAs to enable the formation of the complete dimeric complex. The RNA was then incubated at different temperatures as indicated in Figure 7Aii, fractionated on a native gel, and subjected to Northern analysis with either the U4 or the U6 probes (Figure 7Aii). In the presence of U4 AB oligo, we observed both the monomeric U4 complex and dimeric U4/U6 (Supplementary Figure S10A). Next, we examined the melting temperature of the U4/U6 complex formed in RNA derived from either un-induced or cells silenced for *CBF5*. The results show that the melting temperature of U4/U6 was decreased as a result of *CBF5* silencing (Figure 7Aii-iii, Supplementary Figure S10A-B). Upon *CBF5* silencing, most of the U4/U6 duplex dissociated at 68°C, whereas in un-induced cells the duplex was stable at this temperature. The experiments were repeated three times and statistics was performed comparing the amount of U4/U6 dimer and U4 monomer before and after *CBF5* silencing (Figure 7Aiii). These results suggest that the Ψs contribute to *T. brucei* U4/U6 complex stability, and are mediated by H/ACA snoRNAs.

It is of note that modifications and hypermodifications also exist in loop regions of snRNA that are not known to be involved in base-pairing. For instance, the stem loop of U4 is engaged in binding the protein factors, SNU13 and PRP31 (31), whereas the stem loop IV of U2 is the binding site of U2B'' and U2A’ (32). Of special interest is the presence of Ψs in the Sm site of U2 and U4 snRNAs (see Discussion).

The presence of many Ψs at protein binding sites is a novel unexplored aspect of this modification. To further gain insight into this intriguing finding, we investigated the role of Ψs on U2B'' or U2A’ protein interaction with U2 snRNA using a sensitive microfluidics system (26). To this end, we synthesized Cy5 labelled U2 snRNA that was fully modified by incorporating pseudouridine, or containing regular uridine. Next, we used a microfluidics system in which the U2B'' or U2A’ proteins were bound to the matrix, incubated with the different RNAs, and upon extensive washing the fluorescence of the protein-bound RNA was measured. We then determined the dissociation constant (Kd) of the U2B'' or U2A’ proteins to *T. brucei* U2 snRNA with or without Ψs at 27°C and 37°C (Figure 7Bi-ii, Supplementary Figure S10C). A significant reduction in *K*_d_ was observed for U2B"-U2 snRNA carrying Ψ (*K*_d_ = 104.18 pM), compared to U2B"-U2 snRNA with U instead (*K*_d_ = 317.5 pM) but only at 37°C, suggesting that Ψ strengthens the RNA-protein interaction mainly at an elevated temperature. Similarly, U2A’ had stronger affinity towards pseudouridylated U2 snRNA (*K*_d_ = 1030 pM) compared to RNA with Us (*K*_d_ = 1618 pM) at 37°C (Supplementary Figure S10C). The effect is likely to be general to all protein–Ψ interactions and not restricted to the two tested proteins.

## DISCUSSION

In this study, we demonstrate that *T. brucei* possess the most extensive repertoire of Ψs on U snRNAs described to date, surpassing even the Ψs in mammalian snRNAs. The Ψs are not only present in domains involved in RNA–RNA interactions, but also in domains that are involved in binding of proteins. This is the first study to address the importance of the Ψ not only for RNA–RNA interactions but also for protein-RNA interactions, mostly at high temperatures. Hypermodification was observed in central positions of snRNAs in BSF parasites, as was previously observed for rRNAs (14). We show that a large number of Ψs on rRNAs (68 Ψ) and spliceosomal snRNAs (at least 42 Ψ) are guided by 83 single hairpin H/ACA snoRNA, suggesting that individual *T. brucei* H/ACA snoRNA hairpin is flexible, and can potentially guide Ψ at more than one site. We demonstrate that while the interactions between the snoRNAs and snRNA are non-conventional, depletion of this snoRNA affected the predicted modification on snRNA.

Genome wide Ψ-seq was not previously reported on small RNAs, and this is the first study to achieve this goal. Since not all modifications are found on each molecule, several biological replicates are necessary to accurately detect all Ψs. Moreover, our Ψ-seq protocol failed to identify Ψs very close to the 3′end of the molecule and could not always detect certain Ψs that are adjacent to each other.

Modifications on spliceosomal snRNAs were characterized in only a handful of organisms (28) including nematodes which, like trypanosomes, process their mRNA by *trans*-splicing (33). Of special interest are the hyper-modifications found in the U2 snRNA BSRR (Figure 2Biii). This region is only partially modified in yeast, at position Ψ35 by Pus7p, position Ψ42 by snR81, and position Ψ44 by Pus1 (28). In mammals, six Ψs exist in the BSRR and its vicinity, all guided by scaRNAs (28). Trypanosome U2 carries the Ψ33 (equivalent to Ψ35 in yeast); this Ψ interacts with nucleotides next to the pre-mRNA branch point adenosine during pre-mRNA splicing, and was shown to be essential for splicing (34). Ψ33 is capable of altering the structure of the pre-mRNA U2 snRNA duplex, assisting the 2′-OH of the branch point adenosine to initiate the first splicing step (35,36).

In trypanosomes, the role of the U2 sequence in branch-point recognition was studied previously, and it was concluded that the canonical base-pair model cannot explain the observed branch point selection in the trypanosome-system (27). Here, we show that the Ψ33 and Ψ36 exist in trypanosomes (Figure 2Biii), supporting the notion that a base-pair interaction must take place between the U2 and pre-mRNA at the non-canonical BSRR to assist in the attack of branch point A in *trans*-splicing, as well. This interaction is especially important in the BSF stage, in which the Ψs are hypermodified raising the possibility that a better fit for U2 complementarity exists for mRNAs in BSF. Interestingly, positions Ψ32, Ψ35, Ψ46, Ψ47 and Ψ49 are uniquely modified in trypanosomes (Figure 2Biii). It was recently demonstrated that the splicing factor PRP5 ATPase is involved in monitoring the U2 BSRR interaction (37). Although it is not yet clear how trypanosome U2 interacts with the pre-mRNA, the finding that it contains the modifications Ψ41 and Ψ43 (Figure 2Biii) suggests that PRP5 may play a similar role in monitoring the proper U2-pre-mRNA interactions in trypanosomes.

As expected, Ψ modifications were observed in the U2/U6 interaction domain, which is essential for splicing (38) (Figure 2Bii). Interestingly, in humans, only a single position is modified on U4 in the interaction domain with U6 (28), whereas in trypanosomes, seven such positions exist, and indeed, the melting temperature of *T. brucei* U4/U6 is higher than that in yeast (Figure 7A) (39). The data presented in Figure 7A demonstrate that this thermal stability is mediated by the Ψs. The finding that many of the modifications are also found in protein-binding domains (Figure 2B) is surprising, suggesting that Ψs can possibly contribute to RNA-protein interactions. Indeed, this is the case, since U2B'' and U2A’ were demonstrated to bind with higher affinity to U2 snRNA containing Ψs than to un-modified RNA, especially at higher temperature (Figure 7B, Supplementary Figure S10C). In fact, it was previously reported that Ψs affect the binding of Sm protein to the Sm site, most probably because the modification affects backbone rigidity inducing a conformational preference for the C’3-endo sugar conformation (40). Future works should focus on the mechanism underlining the thermal stability these Ψs confer to the RNA-protein interactions, also from the aspect of protein structure and composition.

Interestingly, Sm binding sites of the *T. brucei* spliceosomal snRNAs deviate from the consensus Sm site and are not rich in uridines but carry Ψs, in contrast to the homologous human sites (Figure 2B) (41). Of note is that neither U4 and U2 snRNAs bind a conventional Sm core, but are replaced by *T. brucei*-specific Sm proteins that we termed SSm (42,43). However, the fact that their Sm sites carry Ψs may contribute to the rigidity of the Sm site (40), and hence its ability to bind a non-conventional Sm core. The need for additional rigidity might come from the need to cope with the ability of the spliceosome snRNP to function at higher temperatures in the mammalian host.

In *Drosophila*, several scaRNAs were shown to modify both rRNA and snRNA using unexpected degree of plasticity (44). However, the promiscuous function in trypanosomes is not rare and several *T. brucei* single hairpin H/ACA snoRNAs have at least a dual function. The dual functionality observed here may be more general. Many ‘orphan’ snoRNAs exist in mammals, and these may guide novel substrates including mRNA, possibly using highly flexible pockets (4). Interestingly, the formation of a non-canonical pocket to guide novel sites was also reported in yeast on U2 snRNA under starvation and heat-shock. The yeast snR81 directs this novel stress-induced Ψ on U2 snRNA based on imperfect base-pairing, having two mismatches in the pseudouridylation pocket (9). The concept that functional guiding may occur even with non-perfect base-pairing between the guide RNA and its target, was recently supported by a study showing that a minimum of 8 base pairs is sufficient for RNA-guided pseudouridylation (45). This flexibility can potentially be used to direct Ψs on related but different substrates.

An important mystery that is highlighted in this study is how U2 and U4 snRNA modifications are introduced at neighboring positions by snoRNAs with non-canonical guiding pockets. The key to this enigma may lie in the known ability of Ψ to base-pair with any of the four major bases (46). Thus, the existing Ψ on the U2 or U4 may help to guide the next Ψ, even by non-canonical interactions. Thus, only because the U2 and U4 snRNA are so highly modified, is it possible for the snoRNAs guiding the modification to tolerate non-perfect base pairing. This however makes predicting the guiders of modifications at these positions an even greater challenge.

This study describes the unprecedented degree of Ψ modifications on *T. brucei* spliceosomal snRNAs, which are found not only on RNA interacting domains but also on protein binding sites, underscoring the effect of this modification on protein binding. The strengthening of protein binding to pseudouridylated RNA is likely to be beneficial especially when the parasite propagates in the mammalian host. However, obtaining further support for this notion awaits *in vivo* experiments demonstrating that depletion of individual Ψs on the protein binding site affects the association specifically with U2 snRNA.

This is the first system wherein a change in a single Ψ guided by a single hairpin H/ACA snoRNA is shown to have an effect on growth and *trans*-splicing. However, we can not rule out the possibility that the growth arrest also stems from depleting the modification on rRNA or any other yet uncharacetrized RNA target guided by the same snoRNA. Nevertheless, the finding that *MTAP* silencing exclusively affects growth and *trans*-splicing demonstrates the importance of this modification for the splicing reaction. The high level of Ψs found on both rRNAs (14) and snRNAs may not only be used to cope with the elevated temperature in the mammalian host but also for overcoming innate immunity during infection since pseudouridylation was shown to affect the innate immune signaling (47).

## DATA AVAILABILITY

The small RNA Ψ-seq and small RNA interactome sequencing data have been deposited in the NCBI SRA database under the accession number PRJNA476671.

## Supplementary Material

gkz477_Supplemental_Files
