# Supplementary material for: Pseudouridines on Trypanosoma brucei spliceosomal small nuclear RNAs and their implication for RNA and protein interactions
Source: Nucleic Acids Res. 2019 May 31;47(14):7633–47. doi: 10.1093/nar/gkz477 (PMC6698659; doi:10.1093/nar/gkz477)
Supplement: gkz477_Supplemental_Files [file gkz477_supplemental_files.zip › Rajan et al., NAR 2019 Supplementary Files_.pdf]

Supplemental Table T1. List of oligonucleotides used in the current study.

**Oligonucleotides used for primer extension**	
U2_snRNA	5'-TGATAAGAACAGTTTAATAA-3'
U2_snRNA (2)	5'-GGACAGCCAACAGTTTTGATCCTT-3'
U2_snRNA (3)	5'-TGATAAGAACAGTTTAATAA-3'
U4_snRNA	5'-CAAACTTTCCCCGAAGGA-3'
U6 snRNA	5'-GCTATATCTCTCGAAGATTG-3'
rRNA_H69	5'-CTCATTAATCCATTCATGCGCGTCTCTAATT-3'
**Oligonucleotides used for U4/U6 duplex analysis**	
U4_oligo_A	5'-CCTCCCTGCGCAAGGCTT-3'
U4_oligo_B	5'-TACCGGATATAGTATTGCAC-3'
U6	5'-CTCTGTTGAATTTCCAGTTTGT-3'
**Oligonucleotides used for MTAP N-terminal YFP construct**	
MTAP_F_HindIII	5'-CCCAAGCTTCCATGCCAGCAAAACGCAAAGG-3'
MTAP_R_NotI	5'-CCCGCGGCCGCCTAGACTCCACACAGTTGCC-3'
**Oligonucleotides used for MTAP antibody preparation**	
MTAP_F_C-TERM_Ab_NheI	5'-AAAGCTAGCCCAACAGTTGGTCATCTCGTCTACGCCGCGGGCCGATTGGGGGAAAATCG-3'
MTAP_R_C-TERM_Ab_HindIII	5'-AAAAAGCTTGACATTGAAAATAGCAGCACCAAGGGTGGAGGTGGAAACACCAGCTCCTT-3'
MTAP_F_N-TERM_Ab_NheI	5'-AAAGCTAGCATGCCAGCAAAACGCAAAGGCAAGATTGCAACGCCTCCGGTAAGCATATT-3'
MTAP_R_N-TERM_Ab_HindIII	5'-AAAAAGCTTCTAGACTCCACACAGTTGCCCTCACGAAGCACATCATAAAGTCTGATGTG-3'
**Oligonucleotides used for probe preparation**	
SLA1_F	5'-AAAGCTCTTTTATGTAGTGTGCGTA-3'
SLA1_R_T7	5'-TTAATACGACTCACTATAGGGAGAGAGTCTCGCTCTCCAGTTTC-3'
TB9Cs4H2_F	5'-TAGGCCCGCCAGCTACCACG-3'
TB9Cs4H2_R_T7	5'-TTAATACGACTCACTATAGGGAGATTTCTTGGCCCGGTAGAACG-3'
TB9Cs4H1_F	5'-TAAGGTTGCCTGTGTACCTC-3'

TB9Cs4H1_R_T7	5'-TTAATACGACTCACTATAGGGAGACAGTAAAGCAAGCCGACGAGG-3'
TB7Cs3H1_F	5'-AAACGACGAAGCTTACCGCG-3'
TB7Cs3H1_R_T7	5'-TTAATACGACTCACTATAGGGAGAGGTGCATGCAAACACACAGC-3'
MTAP_R_T7	5'-TTAATACGACTCACTATAGGGAGACAACTGTTGGATGGGGAAGT-3'
TB6Cs1H2_F	5'-CGAGCCCCGTGGGTGAGGCG-3'
TB6Cs1H2_R_T7	5'-TTAATACGACTCACTATAGGGAGAAGCTCTCGCGGGTCCGATTG-3'
TB9Cs1H1_F	5'-ACAGCACAGAAAATGAAGCT-3'
TB9Cs1H1_R_T7	5'-TTAATACGACTCACTATAGGGAGAGCATCTCGCACAGTGTCGGC-3'
TB11Cs2C2_F	5'-AAGTGATTGACACCTAGGCC-3'
TB11Cs2C2_R_T7	5'-TTAATACGACTCACTATAGGGAGAGATCAGTCAGGGCATAAAAT-3'
TB11Cs2C1_F	5'-TGAATGATGACTGACAAAAC-3'
TB11Cs2C1_R_T7	5'-TTAATACGACTCACTATAGGGAGAAATCAGTCTGGGGCGACAAT-3'
TB6Cs1H1_F	5'-GGCTAGCGAAAACACAGGCG-3'
TB6Cs1H1_R_T7	5'-TTAATACGACTCACTATAGGGAGAAACTCTCGGGTAGCACAGGT-3'
TB11Cs6H1_F	5'-TTTGTCTACGTGTGACTTCA-3'
TB11Cs6H1_R_T7	5'-TTAATACGACTCACTATAGGGAGA ATATCTCTCTCACATGACTC-3'
TB10Cs4H4_F	5'-CTGAGGAGGCCCGTAGCCAC-3'
TB10Cs4H4_R_T7	5'-TTAATACGACTCACTATAGGGAGAAACATAACCATAGCACATGTG-3'
Tb7Cs3H2_F	5'-CTTTCCGCGTGGAGGCTCGG-3'
Tb7Cs3H2_R_T7	5'-TTAATACGACTCACTATAGGGAGAGCATCTCTGACGCCTTTTGT-3'
U4_F	5'-AAGCCTTGCGCAGGGAGGTG-3'
U4_R_T7	5'-TTAATACGACTCACTATAGGGAGA CAAACTTTCCCCGAAGGA-3'
U6_F	5'-GAGCCCTTCGGGGACATCCA-3'
U6_R_T7	5'-TTAATACGACTCACTATAGGGAGAGCTATATCTCTCGAAGATTGACATCAGCC-3'
U3_F	5'-AAGACCGTACTCTGAACAGA-3'
U3_R_T7	5'-TTAATACGACTCACTATAGGGAGAGGATCCTTCTGGAACCGGCT-3'
7SL_F	5'-TTGCTCTGTAACCTTCGGGG-3'
7SL_R_T7	5'-TTAATACGACTCACTATAGGGAGACCGCCTCGCGACGACACTTG-3'
**Oligonucleotides used for stem-loop RNAi construct**	
MTAP_F_Xba	5'-CCATCTAGATTAAGGAGGGCGATGAGTCT-3'
MTAP_R_MluI	5'-CCAACGCGTCAACTGTTGGATGGGGAAGT-3'

MTAP_R_HindIII	5’-CCTAAGCTTCAACTGTTGGATGGGGAAGT-3’
TB11Cs6H1_F_Xba1	5’-AAATCTAGATGTTTAGCCTCTATACAATAG-3’
TB11Cs6H1_R_HindIII	5’-TTTAAGCTTCTTATCCCACATGTATTCTA-3’
**Oligonucleotides used for U2B” protein expression**	
U2B”_cMyc_5'_tag	5'-CTCGAGAATTCGCCACCATGGAACAAAAATTGATAAGTGAGGAAGATGGTGAGCCAAAACAGACGCTTTACATA-3'
U2B”_HIS_3'tag	5'-GTAGCAGCCTGAGTCGTTATTAGTGATGGTGATGGTGATGCCCCGCTTGCGTGTCCCGTCTCCTCTTCCC-3'
**Oligonucleotides used for U2A’ protein expression**	
U2A’_cMyc_5'_tag	5'-CTCGAGAATTCGCCACCATGGAACAAAAATTGATAAGTGAGGAAGATAGGCTGACTCTTGACACCATCAGACGGGCA-3'
U2A’_HIS_3'tag	5'-GTAGCAGCCTGAGTCGTTATTAGTGATGGTGATGGTGATGTGATGTGCGAGTCTTCTTTGTCCCTTTAGA-3'

Figure S1

snRNA	nt	PCF 1	BSF 1	PCF 2	BSF 2	PCF 3	BSF 3	PCF 4	PCF 5	PCF 6	PCF 7	PCF 8	PCF 9	PCF 10	PCF 11	No. of libraries detected	Final Ψ-seq calling	Primer extension mapping	Final Ψ calling
U1	9	+	-	+	+	-	-	+	-	+	-	-	-	-	+	5	Y	NA	Y
U1	44	-	-	-	-	-	-	-	-	-	-	-	-	-	-	NA	-	Y	Y
U1	49	-	-	-	-	-	-	-	-	-	-	-	-	-	-	NA	-	Y	Y
U1	50	-	-	-	-	-	-	-	-	-	-	-	-	-	-	NA	-	Y	Y
U2	7	+	+	-	-	+	+	-	-	+	-	+	-	-	-	6	Y	NA	Y
U2	9	+	+	+	+	+	+	+	+	+	+	+	-	-	+	12	Y	NA	Y
U2	14	+	+	+	+	+	+	+	+	+	+	+	+	-	+	13	Y	NA	Y
U2	16	+	+	+	+	+	+	+	+	+	+	+	+	-	+	13	Y	NA	Y
U2	17	+	+	+	+	-	+	+	+	+	+	+	+	-	+	12	Y	NA	Y
U2	18	+	+	+	+	-	+	+	+	+	+	+	+	-	+	12	Y	NA	Y
U2	22	+	+	+	+	-	+	+	+	+	+	+	+	-	+	12	Y	NA	Y
U2	27	-	-	+	+	-	+	-	-	+	-	-	+	-	-	5	Y	NA	Y
U2	32	+	+	+	+	-	+	+	+	+	+	+	+	-	+	12	Y	NA	Y
U2	33	-	+	+	+	-	+	+	+	+	+	+	+	-	-	10	Y	NA	Y
U2	35	-	-	+	+	-	+	-	+	+	+	-	+	-	-	7	Y	Y	Y
U2	36	-	-	-	-	-	-	-	+	+	+	+	+	+	-	6	Y	Y	Y
U2	41	-	+	+	+	-	+	+	+	+	+	+	+	+	+	12	Y	Y	Y
U2	43	+	+	+	+	+	+	+	+	+	+	+	+	-	+	13	Y	Y	Y
U2	44	-	+	+	+	-	+	+	+	+	+	+	+	-	+	11	Y	Y	Y
U2	46	-	+	+	+	-	+	+	+	+	+	+	+	-	+	11	Y	Y	Y
U2	47	+	+	+	+	-	+	+	-	-	-	-	-	-	-	6	Y	Y	Y
U2	55	+	+	+	+	+	+	-	+	+	+	+	+	-	+	12	Y	Y	Y
U2	59	+	+	+	+	+	+	+	+	+	+	+	+	-	+	13	Y	Y	Y
U2	62	+	+	+	+	+	+	+	+	+	+	+	+	-	+	13	Y	Y	Y
U2	65	+	+	+	+	+	+	+	+	+	+	+	+	-	+	13	Y	Y	Y
U2	74	+	+	+	+	+	+	+	+	+	+	+	+	-	+	13	Y	NA	Y
U2	75	+	+	+	+	+	+	+	+	+	+	+	+	-	+	13	Y	NA	Y
U2	93	+	+	+	+	-	-	+	+	+	+	+	+	-	+	11	Y	Y	Y
U2	95	+	+	+	+	-	-	+	+	+	+	+	+	-	+	11	Y	Y	Y
U2	96	+	+	+	+	-	-	+	+	+	+	+	+	-	+	11	Y	Y	Y
U2	100	+	+	+	+	-	-	+	-	+	+	+	+	-	-	9	Y	Y	Y
U2	102	-	-	-	-	-	-	-	-	-	-	-	-	-	-	NA	-	Y	Y
U2	109	-	-	-	-	-	-	-	-	-	-	-	-	-	-	NA	-	Y	Y
U2	110	-	-	-	-	-	-	-	-	-	-	-	-	-	-	NA	-	Y	Y
U2	112	-	-	-	-	-	-	-	-	-	-	-	-	-	-	NA	-	Y	Y
U2	113	-	-	-	-	-	-	-	-	-	-	-	-	-	-	NA	-	Y	Y
U2	120	-	-	-	-	-	-	-	-	-	-	-	-	-	-	NA	-	Y	Y
U2	121	-	-	-	-	-	-	-	-	-	-	-	-	-	-	NA	-	Y	Y
U4	7	-	+	+	+	-	-	+	+	+	+	+	+	-	+	10	Y	NA	Y
U4	19	+	+	+	+	-	+	+	-	+	+	+	+	-	+	11	Y	Y	Y
U4	21	+	+	+	+	+	+	+	-	+	+	+	+	-	+	12	Y	Y	Y
U4	40	+	+	+	+	-	+	+	-	+	+	+	+	-	+	11	Y	Y	Y
U4	43	+	+	+	+	-	-	+	-	+	+	+	+	-	+	11	Y	Y	Y
U4	44	+	+	+	+	+	+	+	+	+	+	+	+	-	+	13	Y	Y	Y
U4	46	+	+	+	+	-	-	+	-	+	+	+	+	-	+	10	Y	Y	Y
U4	47	+	+	+	+	-	-	-	-	-	-	-	-	-	-	4	-	Y	Y
U4	51	+	+	+	-	-	-	-	-	-	-	-	-	-	-	3	-	Y	Y
U4	54	+	+	+	+	-	-	+	-	+	-	-	+	-	+	8	Y	Y	Y
U4	59	+	+	+	+	-	-	+	+	+	+	-	+	-	+	10	Y	Y	Y
U4	62	+	+	+	+	-	-	+	+	+	+	+	+	-	+	11	Y	Y	Y
U4	64	+	+	+	+	-	-	+	+	+	+	-	-	-	-	8	Y	Y	Y
U4	66	-	+	-	-	-	-	-	-	-	-	-	-	-	-	1	-	Y	Y
U5	40	-	-	-	-	-	-	-	-	-	-	-	-	-	-	NA	-	Y	Y
U5	41	-	-	-	-	-	-	-	-	-	-	-	-	-	-	NA	-	Y	Y
U6	8	-	+	+	+	+	+	+	-	+	-	+	-	-	+	9	Y	Y	Y
U6	17	-	-	-	+	+	+	+	-	-	-	-	-	-	-	4	-	Y	Y
U6	26	-	-	-	-	-	+	+	-	-	-	-	-	-	-	2	-	Y	Y
U6	33	-	+	-	+	-	+	-	-	+	-	-	+	-	+	6	Y	Y	Y
U6	46	-	-	-	-	+	+	+	-	-	-	-	-	-	-	3	-	Y	Y
U6	47	+	+	+	+	+	+	+	-	+	-	+	+	-	+	11	Y	Y	Y
U6	55	-	-	-	-	-	-	-	-	-	-	-	-	-	-	NA	-	Y	Y

Supplementary Figure S1. **Summary of Ψs in *T. brucei* snRNA.** The Ψs called based on the small RNA Ψ-seq protocol on snRNAs (fourteen independent replicates of PCF and BSF samples), and primer extension mapping are indicated by “+”. Regions not analyzed in this study are indicated as “NA”.

Figure S2

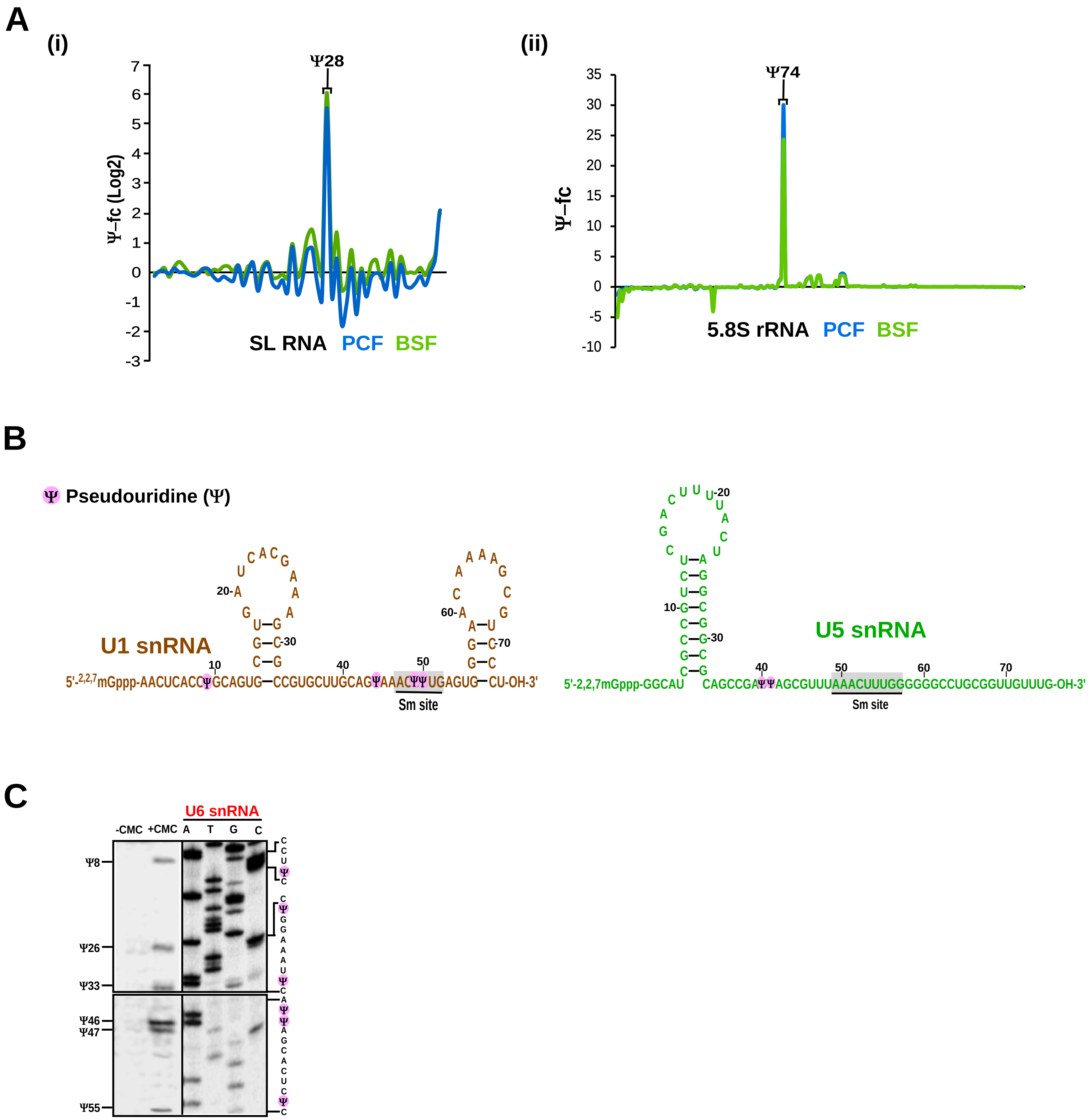
A
(i)
Ψ28
Ψ-fc (Log2)
SL RNA PCF BSF
(ii)
Ψ74
Ψ-fc
5.8S rRNA PCF BSF
B
Ψ Pseudouridine (Ψ)
U1 snRNA
5'-2,2,7mGppp-AACUCACCΨGCAGUG—CCGUGCUUGCAGΨAAACΨΨUGAGUG—CU-OH-3'
Sm site
U5 snRNA
5'-2,2,7mGppp-GGCAU CAGCCGAΨΨAGCGUUUAAACUUUGGGGGGCCUGCGGUUGUUUG-OH-3'
Sm site
C
U6 snRNA
-CMC +CMC A T G C
Ψ8
Ψ26
Ψ33
Ψ46
Ψ47
Ψ55

Supplementary Figure S2. (A) The position of Ψs based on small RNA Ψ-seq for SL RNA and 5.8S rRNA. Line-graph of the Ψ-fc(log2) for PCF and BSF RNAs are presented. (**B) The position of Ψs on U1 and U5 snRNA.** Scheme depicting the position of Ψs based on small RNA Ψ-seq and primer extension are shown on the secondary structure of snRNAs, highlighting functional domains. **(C) Validation of Ψs in *T. brucei* U6 snRNA.** Total RNA (100μg) treated with CMC (+CMC) or untreated (-CMC) was subjected to primer extension and analyzed on a 12% polyacrylamide gel (7M urea). The results along with DNA sequencing performed using the same primer is presented for U6 snRNA. The position of the Ψs are indicated (one nt after the actual stop seen in the gel), as well as the RNA sequence. Differentially contrast adjusted blots are presented.

Figure S3

snRNA	nt	BP	BSF vs PCF Rep1	BSF vs PCF Rep2	BSF vs PCF Rep 3	Hyper
U1	9	Ψ	0.80	0.55	N/A	-
U2	7	Ψ	0.97	1.26	1.25	-
U2	9	Ψ	1.18	1.25	1.20	-
U2	14	Ψ	1.05	0.96	1.09	-
U2	16	Ψ	1.23	1.44	1.43	Y
U2	17	Ψ	1.17	1.93	1.75	Y
U2	18	Ψ	1.26	1.43	1.75	Y
U2	22	Ψ	1.55	1.34	1.85	Y
U2	27	Ψ	1.47	1.94	1.75	Y
U2	32	Ψ	1.49	1.42	1.71	Y
U2	33	Ψ	1.41	0.94	1.47	Y
U2	35	Ψ	1.64	1.82	2.20	Y
U2	36	Ψ	0.04	0.83	1.80	-
U2	41	Ψ	1.80	1.62	1.64	Y
U2	43	Ψ	1.63	1.88	1.35	Y
U2	44	Ψ	1.81	1.82	1.52	Y
U2	46	Ψ	1.73	2.03	1.56	Y
U2	47	Ψ	1.38	1.43	1.50	Y
U2	55	Ψ	1.35	0.95	0.82	-
U2	59	Ψ	1.41	1.46	1.00	Y
U2	62	Ψ	1.44	1.41	1.12	Y
U2	65	Ψ	1.13	0.98	0.97	-
U2	74	Ψ	1.25	1.32	1.08	-
U2	75	Ψ	1.36	1.59	1.24	Y
U2	93	Ψ	1.36	0.76	0.96	-
U2	95	Ψ	1.50	1.22	1.28	-
U2	96	Ψ	1.43	1.10	1.48	Y
U2	100	Ψ	1.45	1.05	1.17	-
U4	7	Ψ	4.86	0.80	3.21	Y
U4	19	Ψ	1.22	1.02	1.92	-
U4	21	Ψ	1.17	1.06	1.46	-
U4	40	Ψ	1.31	1.19	1.95	Y
U4	43	Ψ	1.25	1.13	1.44	-
U4	44	Ψ	1.21	1.06	1.15	-
U4	46	Ψ	1.20	1.15	1.28	-
U4	47	Ψ	1.19	1.15	1.34	-
U4	51	Ψ	1.17	0.70	1.38	-
U4	54	Ψ	1.14	0.99	1.06	-
U4	59	Ψ	1.25	0.73	1.41	-
U4	62	Ψ	1.26	0.74	1.29	-
U4	64	Ψ	1.29	0.58	1.53	-
U4	66	Ψ	1.47	1.06	1.56	Y
U6	8	Ψ	1.74	1.68	1.31	Y
U6	33	Ψ	N/A	2.34	1.53	Y
U6	47	Ψ	1.91	1.26	1.62	Y

Supplementary Figure S3. The fold-change of Ψs in PCF versus BSF based on Ψ-seq for snRNAs. Ψ-fc(log2) across three independent biological replicates for pseudouridylated sites was calculated for Ψs called in small RNA Ψ -seq and Ψ-fc(log2) >1.3 (BSF/PCF) was considered as hypermodified Ψ.

Figure S4

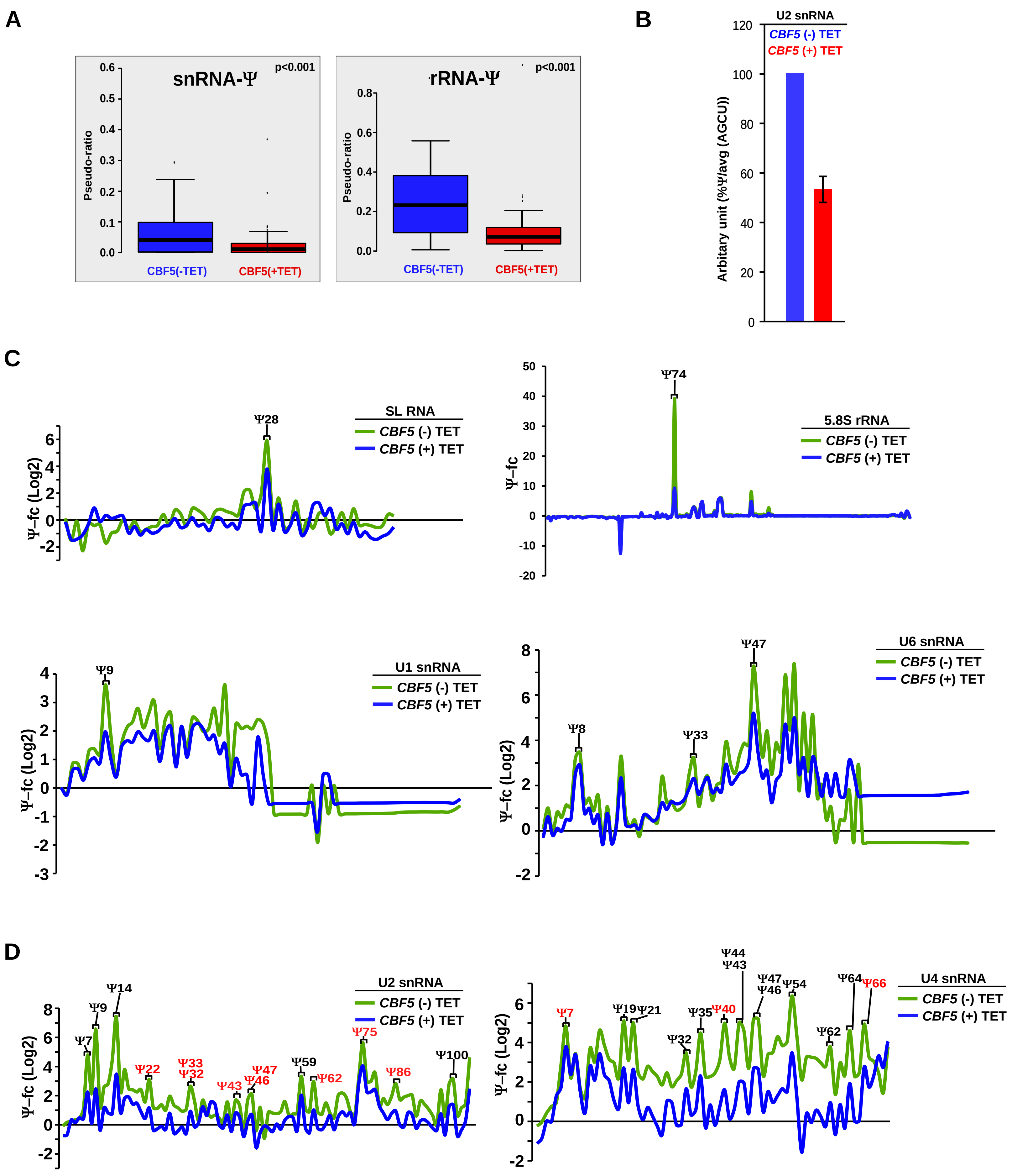

A
snRNA-Ψ
p<0.001
Pseudo-ratio
0.6
0.5
0.4
0.3
0.2
0.1
0.0
CBF5(-TET)
CBF5(+TET)
rRNA-Ψ
p<0.001
0.8
0.6
0.4
0.2
0.0
B
U2 snRNA
CBF5 (-) TET
CBF5 (+) TET
Arbitary unit (%Ψ/avg (AGCU))
120
100
80
60
40
20
0
C
SL RNA
CBF5 (-) TET
CBF5 (+) TET
Ψ28
Ψ–fc (Log2)
5.8S rRNA
Ψ74
Ψ–fc
U1 snRNA
Ψ9
U6 snRNA
Ψ8
Ψ33
Ψ47
D
U2 snRNA
Ψ7
Ψ9
Ψ14
Ψ22
Ψ33
Ψ32
Ψ43
Ψ47
Ψ46
Ψ59
Ψ62
Ψ75
Ψ86
Ψ100
U4 snRNA
Ψ7
Ψ19
Ψ21
Ψ32
Ψ35
Ψ40
Ψ44
Ψ43
Ψ47
Ψ46
Ψ54
Ψ62
Ψ64
Ψ66

Supplementary Figure S4. The effect of *CBF5* silencing on snRNA Ψ. (A) *CBF5* silencing globally affects both rRNA and snRNA Ψs. A quantitative measure of the Ψs on rRNA and snRNA was obtained by calculating the level of stops in the +CMC library before and after CBF5 silencing (Pseudo ratio, y axis). Student's t-test was performed for all Ψs within the library. **(B) Validation of the effect of *CBF5* silencing on U2 snRNA Ψ by mass spectrometry.** Data are represented as mean ± s.e.m. Experiments were done in duplicate. **(C) *CBF5* silencing affects SL RNA, 5.8S rRNA and snRNA Ψs.** Ψ-fc(log2) values (y-axis) were determined for both *CBF5* -TET and +TET based on Ψ-seq libraries. Representative line graphs of indicated RNAs are presented. **(D) Ψ-seq reproducibly detects reduction on Ψs upon CBF5 silencing.** Data from the second replicate is also presented for U2 and U4 snRNA. Ψs hypermodified in BSF are shown in red.

Figure S5

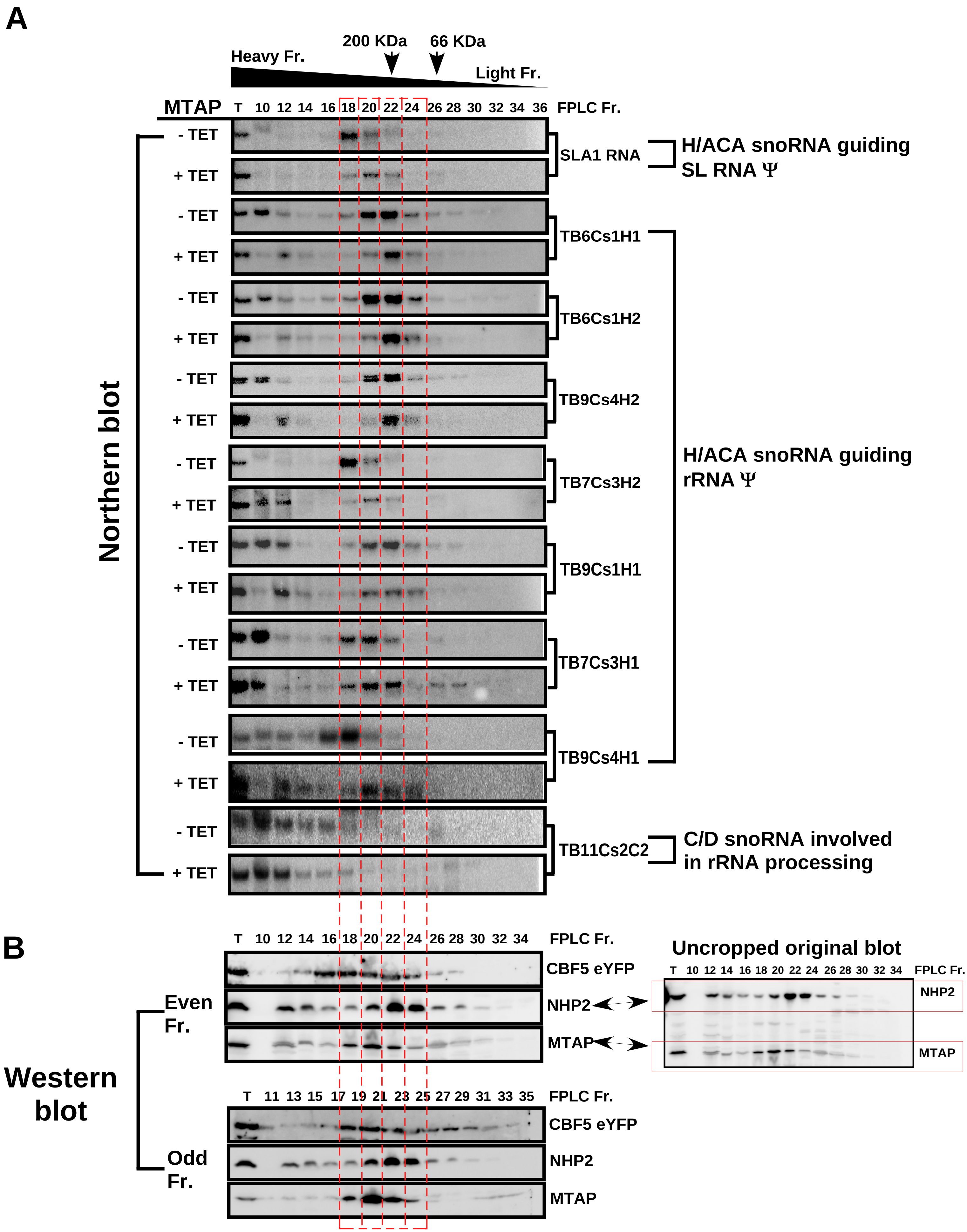

A
Heavy Fr.
200 KDa
66 KDa
Light Fr.
MTAP
T 10 12 14 16 18 20 22 24 26 28 30 32 34 36 FPLC Fr.
Northern blot
- TET
+ TET
SLA1 RNA
H/ACA snoRNA guiding SL RNA Ψ
TB6Cs1H1
TB6Cs1H2
TB9Cs4H2
TB7Cs3H2
TB9Cs1H1
TB7Cs3H1
TB9Cs4H1
H/ACA snoRNA guiding rRNA Ψ
TB11Cs2C2
C/D snoRNA involved in rRNA processing
B
T 10 12 14 16 18 20 22 24 26 28 30 32 34 FPLC Fr.
CBF5 eYFP
NHP2
MTAP
Even Fr.
Western blot
Uncropped original blot
T 10 12 14 16 18 20 22 24 26 28 30 32 34 FPLC Fr.
NHP2
MTAP
T 11 13 15 17 19 21 23 25 27 29 31 33 35 FPLC Fr.
CBF5 eYFP
NHP2
MTAP
Odd Fr.

Supplementary Figure S5. sn(o)RNPs are present in two distinct RNP complexes. (A) Northern analysis. Whole cell extracts from 10^9 uninduced (-TET) or induced (+TET) cells carrying the *MTAP* silencing construct were fractionated on an FPLC Superdex column. Fractions were deproteinated, and RNA was subjected to Northern blot analysis with the indicated RNA probes. The positions of marker proteins BSA (66KDa) and β amylase (200kDa) in the fractionation are indicated by arrows. The heavy and light fractions are indicated. **(B) Western analysis.** FPLC Fractions from wild-type cells harboring C-terminal eYFP tagged *CBF5* were ethanol precipitated and probed with the indicated antibodies, prepared in our lab.

Figure S6

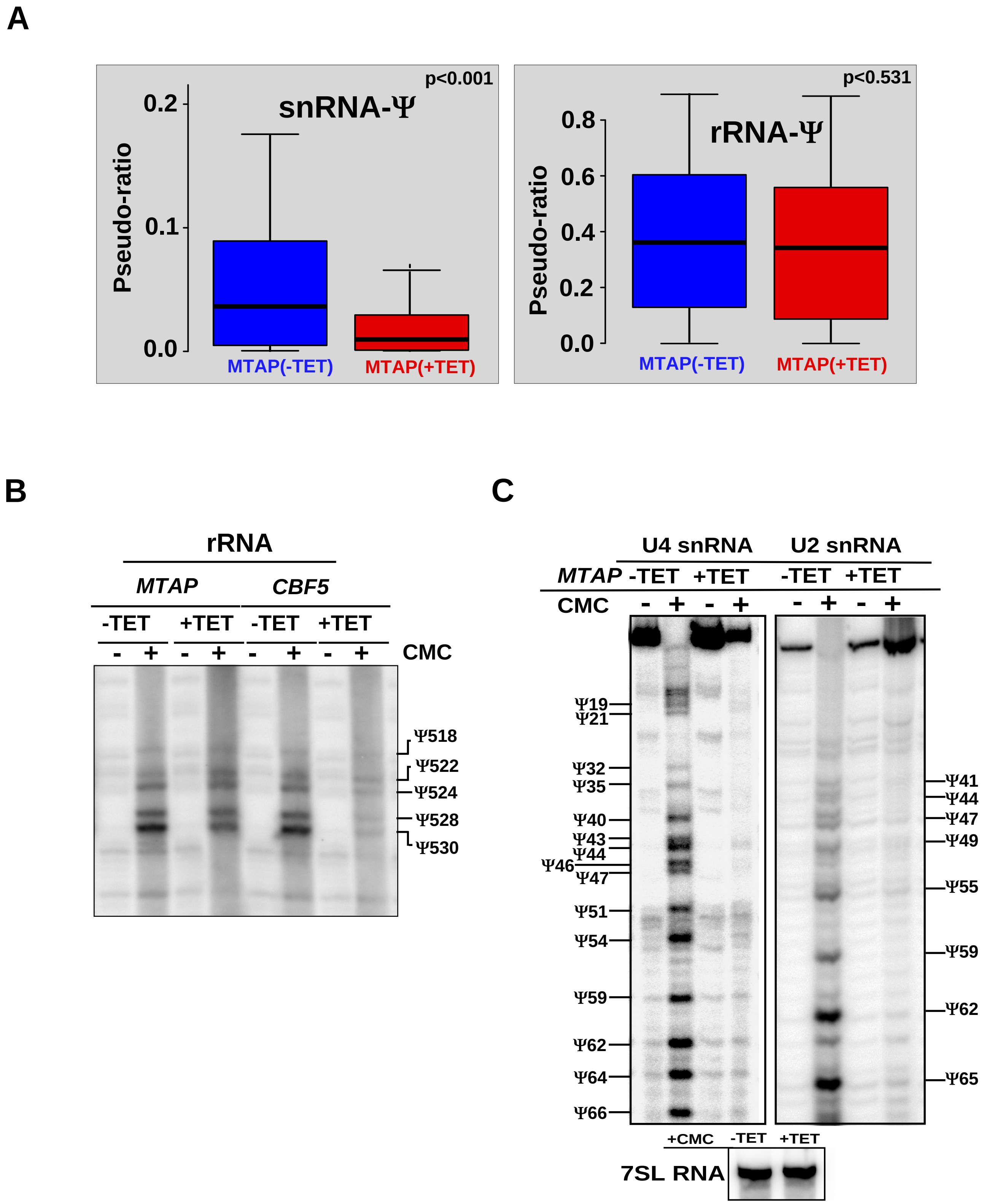
A
p<0.001
snRNA-Ψ
Pseudo-ratio
0.2
0.1
0.0
MTAP(-TET)
MTAP(+TET)
p<0.531
rRNA-Ψ
Pseudo-ratio
0.8
0.6
0.4
0.2
0.0
MTAP(-TET)
MTAP(+TET)
B
rRNA
MTAP
CBF5
-TET
+TET
-TET
+TET
- + - + - + - +
CMC
Ψ518
Ψ522
Ψ524
Ψ528
Ψ530
C
U4 snRNA
U2 snRNA
MTAP -TET +TET -TET +TET
CMC - + - + - + - +
Ψ19
Ψ21
Ψ32
Ψ35
Ψ40
Ψ43
Ψ44
Ψ46
Ψ47
Ψ51
Ψ54
Ψ59
Ψ62
Ψ64
Ψ66
Ψ41
Ψ44
Ψ47
Ψ49
Ψ55
Ψ59
Ψ62
Ψ65
+CMC
-TET
+TET
7SL RNA

Supplementary Figure S6. The effect of *MTAP* silencing on snRNA Ψ. (A) *MTAP* silencing globally affects only snRNA Ψs and not those on rRNA. A quantitative measure of the Ψs on rRNA and snRNA was obtained by calculating the level of stops in the +CMC library before and after CBF5 silencing (Pseudo ratio, y axis). Student's t-test was performed for all Ψs within the library. **(B) Validation of the effect of *MTAP* silencing on rRNA Ψ by primer extension.** (**C) Validation of the effect of *MTAP* silencing on snRNA Ψ by primer extension.** 7SL RNA served as loading control in the +CMC lane.

Figure S7

A

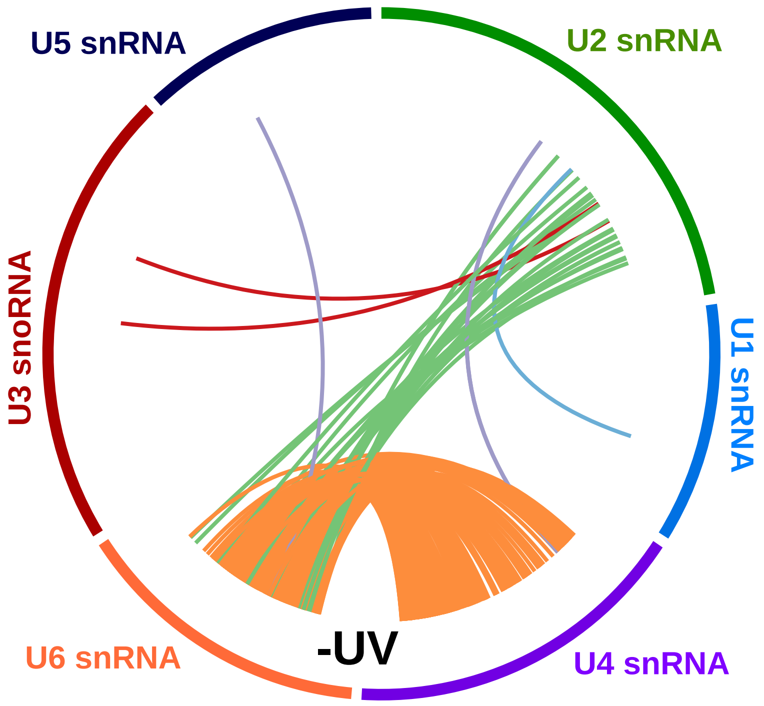

U5 snRNA
U2 snRNA
U3 snoRNA
U1 snRNA
U6 snRNA
-UV
U4 snRNA

B

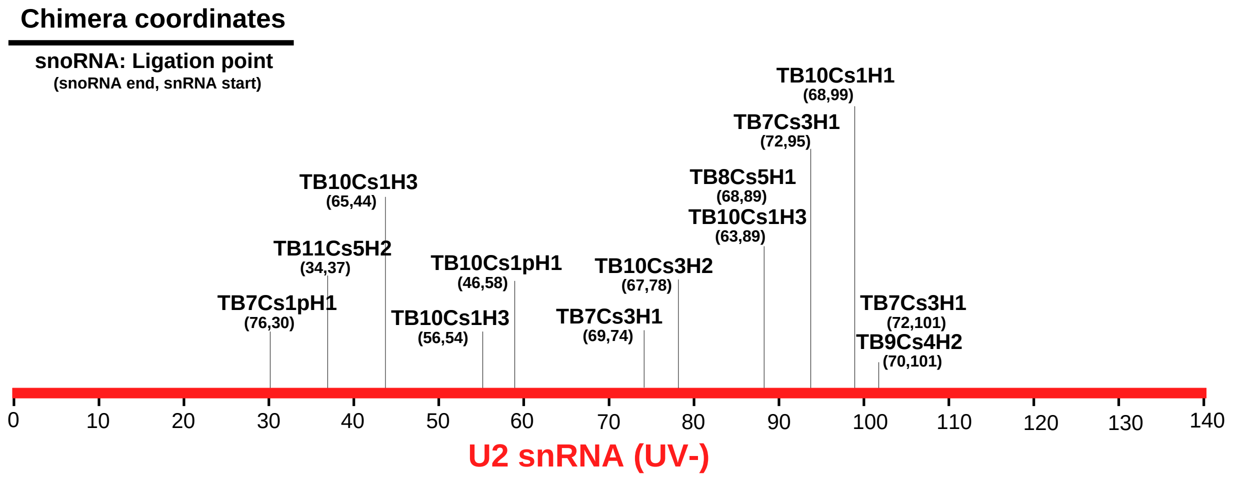

Chimera coordinates
snoRNA: Ligation point
(snoRNA end, snRNA start)
TB10Cs1H1
(68,99)
TB7Cs3H1
(72,95)
TB10Cs1H3
(65,44)
TB8Cs5H1
(68,89)
TB10Cs1H3
(63,89)
TB11Cs5H2
(34,37)
TB10Cs1pH1
(46,58)
TB10Cs3H2
(67,78)
TB7Cs1pH1
(76,30)
TB10Cs1H3
(56,54)
TB7Cs3H1
(69,74)
TB7Cs3H1
(72,101)
TB9Cs4H2
(70,101)
0 10 20 30 40 50 60 70 80 90 100 110 120 130 140
U2 snRNA (UV-)

Supplementary Figure S7. Small RNA interactome. (A) *In vivo* AMT-psoralen UV cross-linking was performed and used to identify interacting RNA molecules upon ligation. RNA (-UV and +UV) was analyzed for intermolecular or intramolecular cross-links. Circos plot representing the interactome of U snRNA is presented for -UV. **(B) Interactions between U2 and snoRNAs in -UV sample.** The ligation points of sn(o)RNA to U2 snRNA along its sequence without UV irradiation are depicted on a linear scale. Coordinates of the resulting chimera [I PREFER "ADDUCTS"] are given as the nucleotide on snoRNAs ligated to the indicated position on U2 snRNA.

Figure S8

A

	UV(-)	UV(+)
Mapped reads	6,060,063	20,676,989
Single matches	6,016,850	20,258,439
Total chimera	43213	418550
Intramolecular chimera	32749	154067
Intermolecular chimera	10464	264483

B

RNA 1	RNA 2	UV-(RPM)	UV+(RPM)
U2	U6	2389	325
U4	U6	20737	1346
SL	SLA1	191	34

C

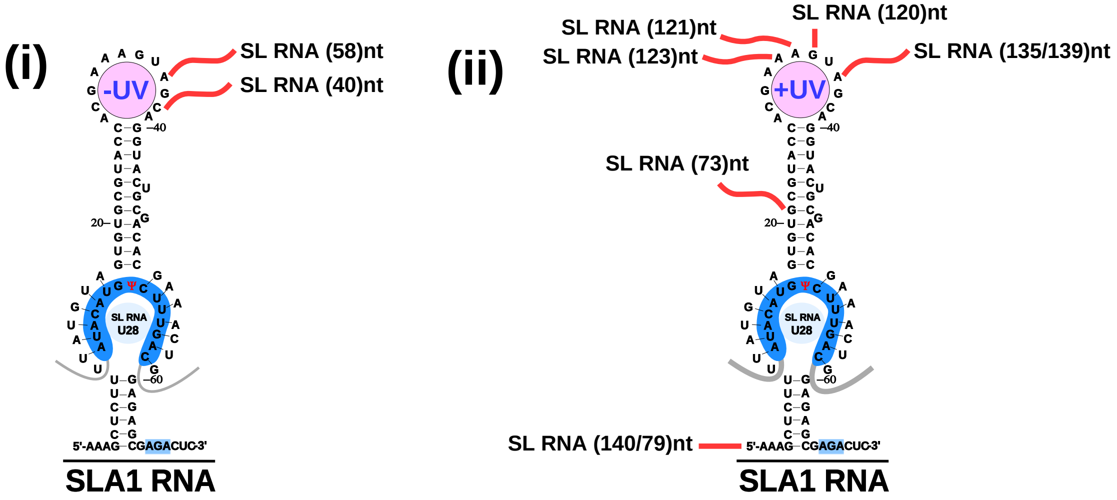

D

	snoRNA	Start	End	rRNA	Start(-1)	End	Predicted position	rRNA Ψ	Distance from Ψ
1	TB11Cs4H2	15	50	rRNA	1972	2007	1978	18s(U61)	6
	TB11Cs4H2	0	42	rRNA	1973	2011	1978	18s(U61)	5
	TB11Cs4H2	14	49	rRNA	1973	2008	1978	18s(U61)	5
	TB11Cs4H2	2	32	rRNA	1974	2026	1978	18s(U61)	4
2	TB10Cs1H3	4	69	rRNA	1975	2005	1957	18s(U40)	-18
	TB10Cs1H3	4	69	rRNA	1978	2018	1957	18s(U40)	-21
	TB10Cs1H3	33	64	rRNA	1980	2039	1957	18s(U40)	-23
3	TB10Cs4H4	32	67	rRNA	2411	2462	2422	18s(U505)	11
4	TB6Cs1H3	1	37	rRNA	2570	2608	2579	18s(U662)	9
	TB6Cs1H3	1	66	rRNA	2574	2612	2579	18s(U662)	5
	TB6Cs1H3	1	67	rRNA	2575	2609	2579	18s(U662)	4
	TB6Cs1H3	12	61	rRNA	2576	2607	2579	18s(U662)	3
	TB6Cs1H3	1	68	rRNA	2577	2608	2579	18s(U662)	2
	TB6Cs1H3	0	68	rRNA	2577	2612	2579	18s(U662)	2
	TB6Cs1H3	1	64	rRNA	2582	2618	2579	18s(U662)	-3
5	TB11Cs3pH1	45	85	rRNA	2791	2861	2859	18s(U942)	68
6	TB9Cs1H1	6	55	rRNA	2935	2975	2913	18s(U996)	-22
7	TB1Cs1H1	35	65	rRNA	3513	3561	3509	18s(U1592)	-4
8	TB8Cs5H1	3	49	rRNA	3936	4003	3962	18s(U2045)	26
9	TB11Cs5H2	4	51	rRNA	4061	4094	4038	18s(U2121)	-23
10	TB9Cs8H1	7	39	rRNA	6444	6481	6447	28s_alpha(U1158)	3
11	TB9Cs2H2	38	71	rRNA	6521	6595	6537	28s_alpha(U1248)	16
	TB9Cs2H2	40	71	rRNA	6522	6604	6537	28s_alpha(U1248)	15
12	TB7Cs3H2	71	101	rRNA	7085	7139	7036	28s_alpha(U1747)	-49
13	TB7Cs1pH1	35	70	rRNA	8115	8155	8129	28s_beta(U522)	14
	TB7Cs1pH1	40	74	rRNA	8138	8175	8129	28s_beta(U522)	-9
14	TB9Cs1ppH1	10	76	rRNA	8222	8271	8218	28s_beta(U611)	-4
15	TB8Cs2H1A	38	68	rRNA	8918	8961	8907	28s_beta(U1300)	-11
16	TB9Cs1H1	12	68	rRNA	8920	8956	8941	28s_beta(U1334)	21
17	TB6Cs1H4	41	78	rRNA	8981	9019	9016	28s_beta(U1409)	35
18	TB6Cs1H2	0	47	rRNA	9043	9074	8984	28s_beta(U1377)	-59
	TB6Cs1H2	0	47	rRNA	9043	9074	8984	28s_beta(U1377)	-59
19	TB9Cs4H2	41	71	rRNA	9064	9102	9005	28s_beta(U1398)	-59

Supplementary Figure S8. Small RNA interactome. (A) The total number of reads in the RNA (-UV or +UV) forming intermolecular or intramolecular cross-links was analyzed, and the numbers of reads are presented. **(B)** RPM of reads obtained U2-U6, U4-U6 and SLA1-SL RNA from UV treated and untreated RNA. **(C)** Ligations obtained from SLA1-SL RNA are shown across the secondary structure of SLA1 RNA. SLAI RNA guides Ψ28 in SL RNA. (i) Ligations of untreated (-UV) RNA depicting ligations within the epical loop of SLA1 RNA. The nucleotide of SL RNA ligated to SLA1 RNA is shown in parenthesis for each SLRNA. (ii) Ligation from +UV RNA depicting ligations not only within the epical loop of SLA1 RNA, but also close to the pseudouridylation pocket. The nucleotide of SL RNA ligated to SLA1 RNA is shown in parenthesis for each SLRNA. **(D) Ligations obtained for H/ACA snoRNA-rRNA in the small RNA interactome.** The reads in the RNA (+UV) were analyzed for intermolecular cross-links between H/ACA snoRNA and rRNA. The ligations obtained from 19 H/ACA snoRNA-rRNA interactions are presented. Coordinates of the obtained chimera are indicated as the nucleotide on snoRNAs ligated to the stated position on the rRNA.

Figure S9

	snRNA	snoRNA	UV-(RPM)	UV+(RPM)
1	U2	TB10Cs1H1	95	0
2	U2	TB10Cs1H3	286	0
3	U2	TB10Cs1pH1	95	0
4	U2	TB10Cs3H1	95	3
5	U2	TB10Cs3H2	95	0
6	U2	TB10Cs-7H1	0	3
7	U2	TB11Cs4H1	0	3
8	U2	TB11Cs4H2	0	7
9	U2	TB11Cs5H1	0	3
10	U2	TB11Cs5H2	95	0
11	U2	TB6Cs1H1	0	3
12	U2	TB6Cs1H2	0	3
13	U2	TB6Cs1H3	0	3
14	U2	TB7Cs1pH1	95	11
15	U2	TB7Cs3H1	286	7
16	U2	TB7Cs3H2	0	3
17	U2	TB8Cs3H-1	3631	211
18	U2	TB8Cs4H2	0	3
19	U2	TB8Cs5H1	95	7
20	U2	TB9Cs1H1	0	18
21	U2	TB9Cs1ppH1	0	3
22	U2	TB9Cs2H2	0	3
23	U2	TB9Cs4H2	191	3
24	U2	TB9Cs4H3	0	3

Supplementary Figure S9. Reads of snoRNA-U2 snRNA chimera from the small RNA interactome. The total number of reads in the RNA (-UV) and (+UV) was analyzed for intermolecular cross-links; reads are presented for ligations between sn(o)RNA and U2 snRNA. The data are presented as RPM for 24 snoRNAs. Reads specific to -UV are highlighted in green, +UV in blue, and those obtained in both samples, in orange.

Figure S10

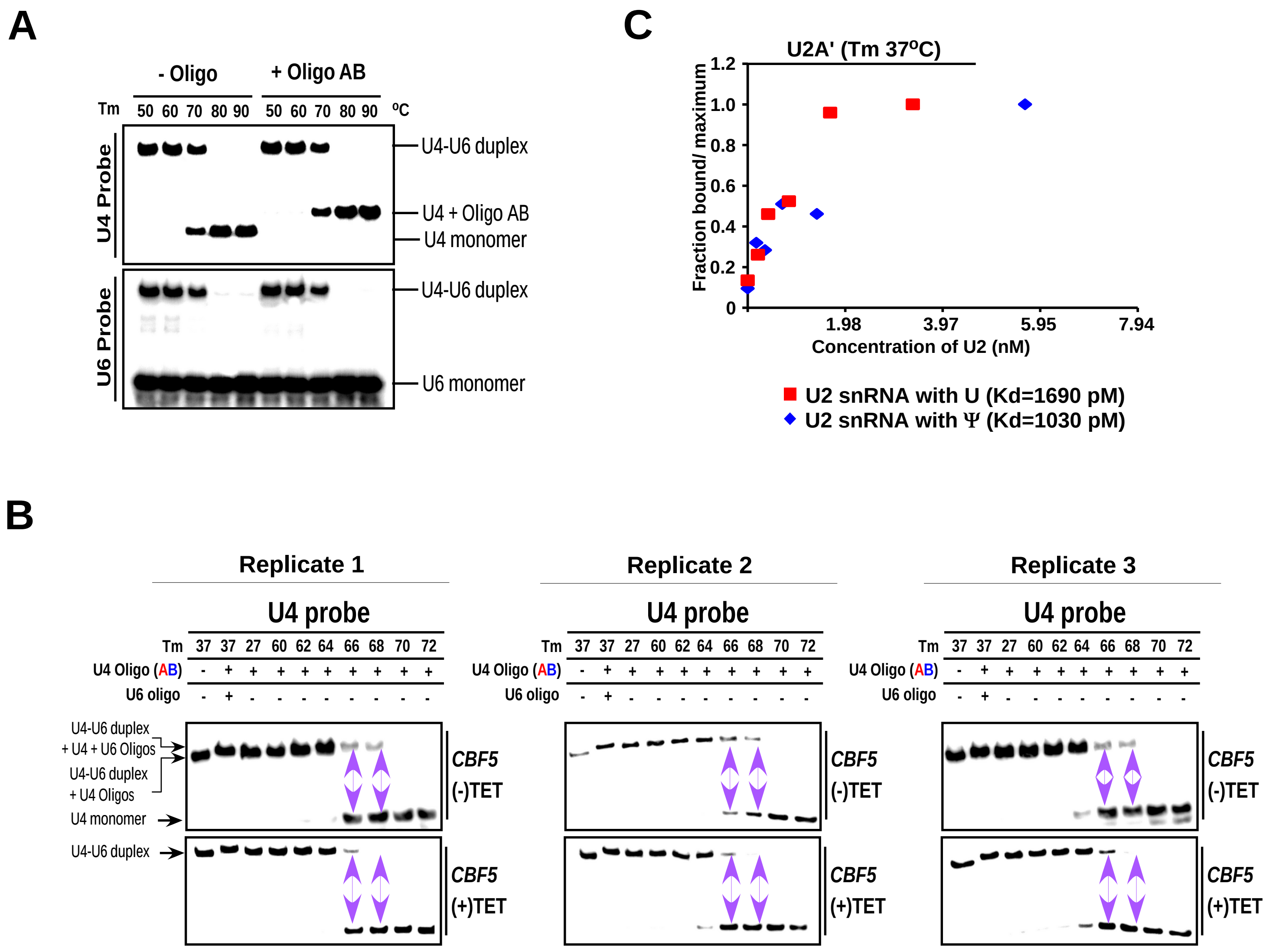
A
- Oligo
+ Oligo AB
Tm 50 60 70 80 90 50 60 70 80 90 °C
U4 Probe
U6 Probe
U4-U6 duplex
U4 + Oligo AB
U4 monomer
U4-U6 duplex
U6 monomer
C
U2A' (Tm 37°C)
Fraction bound/ maximum
1.2
1.0
0.8
0.6
0.4
0.2
0
1.98
3.97
5.95
7.94
Concentration of U2 (nM)
U2 snRNA with U (Kd=1690 pM)
U2 snRNA with Ψ (Kd=1030 pM)
B
Replicate 1
Replicate 2
Replicate 3
U4 probe
Tm 37 37 27 60 62 64 66 68 70 72
U4 Oligo (AB) - + + + + + + + + +
U6 oligo - + - - - - - - - -
U4-U6 duplex + U4 + U6 Oligos
U4-U6 duplex + U4 Oligos
U4 monomer
U4-U6 duplex
CBF5 (-)TET
CBF5 (+)TET

Supplementary Figure S10. *T. brucei* U4/U6 duplex formation. (A) Validation for reconstitution of U4/U6 duplexes from *T. brucei* total RNA. U4/U6 duplexes reconstituted from total RNA derived from WT PCF cells were incubated with or without the indicated oligonucleotide (see Figure 7A) and subjected to different temperatures, as indicated. Annealed RNA was separated on a 12% native gel and Northern analysis was performed with U4 (upper panel) and U6 (lower panel) RNA probe. **(B) Biological replicates indicating that U4/U6 duplex stability depends on its Ψs.** U4/U6 duplexes reconstituted from total RNA derived from cells carrying *CBF5* silencing construct, either uninduced (-TET) or after 2.5 days of silencing (+TET), were incubated with the indicated oligonucleotide and subjected to different temperatures, as indicated. Annealed RNA was separated on a 12% native gel and subjected to Northern analysis with a U4 RNA probe. Data represent results from three independent replicates. **(C) A dose response of Cy5 labeled U2 snRNA binding to U2A' in a microfluidic device.** After the U2A'-U2 snRNA interaction reached equilibrium at 37°C, the free and U2A' bound RNA concentration was measured. The data were normalized to maximum, and affinity was calculated by non-linear least squares fitting.
